# A dominant negative variant of *RAB5B* disrupts maturation of surfactant protein B and surfactant protein C

**DOI:** 10.1073/pnas.2105228119

**Published:** 2022-02-04

**Authors:** Huiyan Huang, Jiehong Pan, David R. Spielberg, Neil A. Hanchard, Daryl A. Scott, Lindsay C. Burrage, Hongzheng Dai, David Murdock, Jill A. Rosenfeld, Ariz Mohammad, Tao Huang, Anika G. Lindsey, Hyori Kim, Jian Chen, Avinash Ramu, Stephanie A. Morrison, Zachary D. Dawson, Alex Z. Hu, Eric Tycksen, Gary A. Silverman, Dustin Baldridge, Jennifer A. Wambach, Undiagnosed Diseases Network, Stephen C. Pak, Steven L. Brody, Tim Schedl

**Affiliations:** ^a^Department of Pediatrics, Washington University School of Medicine, St. Louis, MO 63110;; ^b^Department of Medicine, Washington University School of Medicine, St. Louis, MO 63110;; ^c^Department of Pediatrics, Section of Pulmonary Medicine, Baylor College of Medicine, Houston, TX 77030;; ^d^Department of Molecular and Human Genetics, Baylor College of Medicine, Houston, TX 77030;; ^e^Department of Molecular Physiology and Biophysics, Baylor College of Medicine, Houston, TX 77030;; ^f^Department of Genetics, Washington University School of Medicine, St. Louis, MO 63110;; ^g^Genome Technology Access Center, McDonnell Genome Institute, Washington University School of Medicine, St. Louis, MO 63110

**Keywords:** RAB5B, *Caenorhabditis elegans*, endocytosis, surfactant proteins, surfactant dysfunction disorder

## Abstract

Pathogenic variants in surfactant proteins SP-B and SP-C cause surfactant deficiency and interstitial lung disease. Surfactant proteins are synthesized as precursors (proSP-B, proSP-C), trafficked, and processed via a vesicular-regulated secretion pathway; however, control of vesicular trafficking events is not fully understood. Through the Undiagnosed Diseases Network, we evaluated a child with interstitial lung disease suggestive of surfactant deficiency. Variants in known surfactant dysfunction disorder genes were not found in trio exome sequencing. Instead, a de novo heterozygous variant in *RAB5B* was identified in the Ras/Rab GTPases family nucleotide binding domain, p.Asp136His. Functional studies were performed in *Caenorhabditis elegans* by knocking the proband variant into the conserved position (Asp135) of the ortholog, *rab-5*. Genetic analysis demonstrated that *rab-5*[Asp135His] is damaging, producing a strong dominant negative gene product. *rab-5*[Asp135His] heterozygotes were also defective in endocytosis and early endosome (EE) fusion. Immunostaining studies of the proband’s lung biopsy revealed that RAB5B and EE marker EEA1 were significantly reduced in alveolar type II cells and that mature SP-B and SP-C were significantly reduced, while proSP-B and proSP-C were normal. Furthermore, staining normal lung showed colocalization of RAB5B and EEA1 with proSP-B and proSP-C. These findings indicate that dominant negative–acting RAB5B Asp136His and EE dysfunction cause a defect in processing/trafficking to produce mature SP-B and SP-C, resulting in interstitial lung disease, and that RAB5B and EEs normally function in the surfactant secretion pathway. Together, the data suggest a noncanonical function for RAB5B and identify *RAB5B* p.Asp136His as a genetic mechanism for a surfactant dysfunction disorder.

## Significance

The Rab5 GTPase functions in early endosome (EE) fusion in the endocytic pathway. Here, we propose that RAB5B also has a noncanonical vesicular fusion function in the regulated secretion pathway that produces mature surfactant proteins SP-B and SP-C in the lung. This function was revealed from investigation of a proband with interstitial lung disease suggestive of a surfactant dysfunction disorder who carried a de novo Asp136His variant in the *RAB5B* gene. Our modeling in *C. elegans* provided information on the genetic and cell biological mechanism, and analyses of proband and normal lung biopsies suggested a function for RAB5B and EEs in surfactant protein processing/trafficking. This work indicates that *RAB5B* p.Asp136His causes a surfactant dysfunction disorder.

Rab5, a member of the Ras/Rab superfamily of small GTPases (1, 2), is a master regulator of the endocytic pathway, controlling early endosome (EE) trafficking in animals (3, 4). Guanosine-5'-triphosphate (GTP)-bound Rab5 is active and associated with the EE membrane via its C-terminal isoprenoid modification. In the endocytic pathway, active Rab5 promotes the heterotypic fusion of nascent endocytic vesicles with EEs and the homotypic fusion of cargo-containing EE vesicles with each other. In contrast, guanosine 5′-diphosphate (GDP)-bound Rab5 is inactive and resides in the cytoplasm. Effector proteins promote EE vesicle fusion and cycling between the GTP- and GDP-bound states of Rab5 (5–7). In addition to its canonical endocytic function, Rab5 has noncanonical functions: for example, in promoting fusion of nascent sorting vesicles with EEs in the regulated secretion pathway in mast cells and in the *Drosophila* salivary gland (8, 9).

Lung surfactant is a protein–lipid mixture that lines the air–liquid surface of the lung alveolar epithelium, reducing surface tension to prevent alveolar collapse and enhance lung compliance during breathing (10, 11). There are four major lung surfactant proteins; hydrophobic SP-B and SP-C function in the formation of the protein–lipid matrix that lines the alveolar epithelium, while hydrophilic SP-A and SP-D are collectin family members that function in innate immunity. The regulated surfactant protein secretion pathway in alveolar type II (AT2) cells commences with translation in the endoplasmic reticulum (ER) of precursor proteins proSP-B and proSP-C, which contain an N-terminal propeptide and a C-terminal mature peptide (12–15). proSP-B and proSP-C are trafficked through the Golgi, where they bud into nascent sorting vesicles that progress to the multivesicular body (MVB) and then, transit to the lamellar body. Acidification in the MVB and lamellar body stimulates proteolytic processing to mature SP-B and SP-C and their association with phospholipids. Surfactant protein–lipid complexes are stored in the lamellar body for stimulus-induced secretion. Factors that control the early steps and trafficking of proSP-B– and proSP-C–containing vesicles to the MVB are not well understood (16–18).

Pathogenic variants in the genes that encode SP-B and SP-C (*SFTPB* and *SFTPC*) and genes that are involved in surfactant production (*ABCA3* and *NKX2-1*) result in surfactant dysfunction disorders, one of the genetic causes of interstitial lung disease (ILD) (10, 19–21). Biallelic loss-of-function variants in *SFTPB* result in full-term infants with severe respiratory distress syndrome, which is typically lethal within the first few months after birth (22). In contrast, dominant variants in *SFTPC* result in lung diseases with variable penetrance and expressivity, ranging from neonatal respiratory distress syndrome to ILD in the fifth and sixth decades of life (23). Genetic screening of full-term infants with lethal surfactant dysfunction disorders identified cases that lacked variants in the known causal genes, indicating that there are yet to be identified genes that function in surfactant production (24–26), which is not surprising given the complex biology of the regulated surfactant secretion pathway.

Here, we describe a female child identified through the Undiagnosed Diseases Network (UDN) (27) presenting with ILD suggestive of a surfactant dysfunction disorder, dysmorphic features, and global developmental delay. Trio exome sequencing identified that the proband carried a de novo heterozygous c.406G > C [NM_002868.3], p.Asp136His missense variant in *RAB5B*, a gene not previously associated with disease. We exploited the facile genetics and the well-developed assays for endocytosis and EEs in *Caenorhabditis elegans* (28–30) to assess the functional consequences of the proband’s *RAB5B* variant when modeled in the worm ortholog (*SI Appendix*, Fig. S1). The goals of the functional analysis were to determine if the variant was damaging in vivo, to provide insight into the genetic mechanism of the de novo dominant presentation, and to assess alterations in EE biology. We examined the proband’s lung tissue to understand the basis of the ILD as well as normal donor lung tissue to assess the relationship of *RAB5B* to the regulated surfactant secretion pathway (*SI Appendix*, Fig. S1). Our findings indicate that the *RAB5B* variant disrupts maturation of surfactant proteins B and C, resulting in ILD in the proband, and that RAB5B and EEs function in the regulated lung surfactant secretion pathway.

## Results

### ILD and Developmental Delay in a Proband with a De Novo Variant in *RAB5B*.

**Fig. 1.**
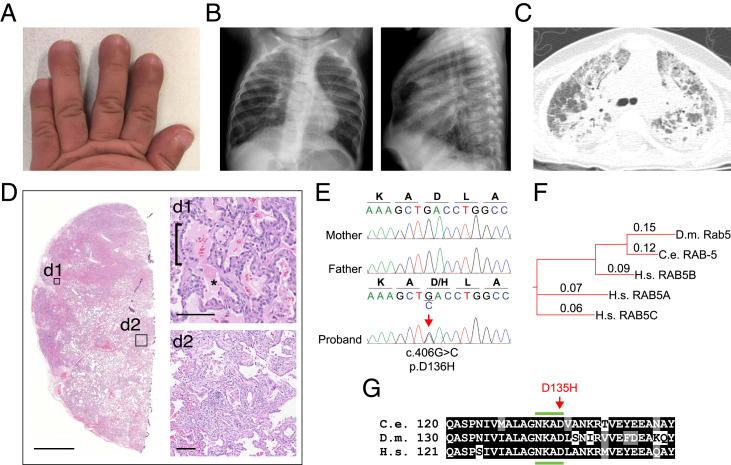
Clinical features of the proband with a de novo variant in *RAB5B*. (*A*) Shortened broad fingers with nail clubbing. (*B*) Hyperinflation of lungs and interstitial opacities by chest radiograph (posterior–anterior and lateral views) at 7 mo of age. (*C*) Computed tomography of the lung at 11 mo of age shows acute and chronic interstitial changes. (*D*) Lung biopsy stained with hematoxylin and eosin (higher-power details are in insets *d1* and *d2*) shows alveolar filling (*d1*; asterisk), AT2 cell hyperplasia (*d1*; bracket), remodeling (*d2*), and fibrosis (*d2*). (Scale bars: *D*, 2.5 mm; *d1* and *d2*, 100 μm.) (*E*) Dideoxy (Sanger) sequencing traces from parents and the proband showing a de novo heterozygous variant in *RAB5B*, c.406G > C, p.Asp136His. (*F*) Phylogenetic tree of Rab5 indicating the evolutionary orthologs and human paralogs. Alignment distance values are shown (Clustal Omega). C.e., *C. elegans*; D.m., *D. melanogaster*; H.s., *Homo sapien*s. (*G*) Alignment of the RAB5B sequence from H.s. residues 121 to 151 with orthologs from indicated species. The location of the variant edited in *C. elegans* corresponding to the conserved aspartate [D] in the proband is indicated (red arrow). The fourth region of the conserved nucleotide binding domain (NKXD) is indicated (green bars above and below). Identical residues are shaded black; conserved residues are gray (*SI Appendix*, Fig. S2).

We identified a female of Pakistani ancestry with global developmental delay, dysmorphic features, and ILD leading to progressive respiratory failure and death at age 2. The proband was born to a 39-y-old mother and a 46-y-old father at 34-wk gestation by Caesarean section and weighed 2.7 kg (91st percentile). She had no respiratory symptoms at birth. Evaluation at 6 mo was significant for hypotonia, a broad nasal bridge with telecanthus, short squared digits, and overlapping second toes. She had profound developmental delay with static encephalopathy. Brain magnetic resonance imaging and electroencephalogram were normal. At 11 mo of age, she had digital clubbing (Fig. 1*A*) and a chest radiograph that showed hyperinflation and diffuse interstitial opacities (Fig. 1*B*). A computed tomography scan of the chest demonstrated diffuse ground glass opacities, areas of consolidation, and peripheral cysts, consistent with ILD and suggestive of a surfactant dysfunction disorder (Fig. 1*C*).

The proband developed chronic tachypnea and had frequent hospitalizations for respiratory distress. At 17 mo of age, she required continuous supplemental oxygen, and a lung biopsy was performed. Histologic examination of lung tissue showed alveolar proteinosis, AT2 cell hyperplasia, lobar remodeling, and early fibrosis, consistent with a chronic disorder of surfactant dysfunction (Fig. 1*D*). Transmission electron microscopy was not performed. Clinical immunostaining of the lung tissue reported normal levels of ABCA3, TTF-1, SP-D, and proSP-C within AT2 cells but diminished SP-B levels. Despite therapies for ILD, the child died at the age of 2 y from respiratory failure.

A chromosomal microarray analysis (Baylor Genetics; CMA-HR+SNP version 11.2) was performed and was nondiagnostic. Trio exome sequencing was performed. No variants, losses, or gains were found in genes known to be involved in surfactant metabolism (*SI Appendix*, Table S1). Instead, a de novo variant in *RAB5B* was identified, corresponding to p.Asp136His (p.D136H, c.406G > C, NM_002868.3), and was subsequently confirmed by Sanger sequencing (Fig. 1*E*). The variant is not found in the sequence database Genome Aggregation Database (gnomAD) of ∼140,000 individuals (https://gnomad.broadinstitute.org; accessed October 2021) (31). The missense change is predicted to be damaging based on a combined annotation dependent depletion (CADD) score of 29.6 and a MutationTaster score of 81 (32, 33). *RAB5B* is likely not a haplo-insufficient gene based on tolerance to heterozygous loss-of-function variants, with an observed to expected ratio of 0.19 to 0.75, and probability of being loss-of-function intolerant (pLI) = 0.03 (gnomAD v2.1.1).

In humans, RAB5 has EE vesicular fusion functions mediated by three paralogous genes *RAB5A*, *RAB5B*, and *RAB5C*, compared with single ancestral orthologs in *C. elegans* (*rab-5*) and *Drosophila melanogaster* (*Rab5*) (34) (Fig. 1*F*), suggesting redundant and unique functions for the paralogs in humans. The *RAB5B* residue p.D136 is highly conserved and resides in the fourth region of the small GTPase nucleotide binding domain (Fig. 1*G* and *SI Appendix*, Fig. S2*A*), indicating that the variant may disrupt nucleotide binding, hydrolysis, and/or release. RAS family GTPases have a nearly identical nucleotide binding domain as RAB family GTPases (35). In a forward genetic screen, dominant negative mutations in the *C. elegans* ortholog of *HRAS*, *let-60*, were identified (36). One of these, D119N (allele *sy93*), is in the fourth region of the nucleotide binding domain of *let-60* at the residue corresponding to human *RAB5B* p.D136 and *C. elegans rab-5* D135 (Fig. 1*G* and *SI Appendix*, Fig. S2 *B* and *C*). These findings led us to propose that the phenotype of the proband results from the de novo heterozygous *RAB5B* p.D136H variant encoding a dominant negative gene product.

### *C. elegans rab-5*[D135H] Is Damaging, Producing a Dominant Negative Gene Product.

**Fig. 2.**
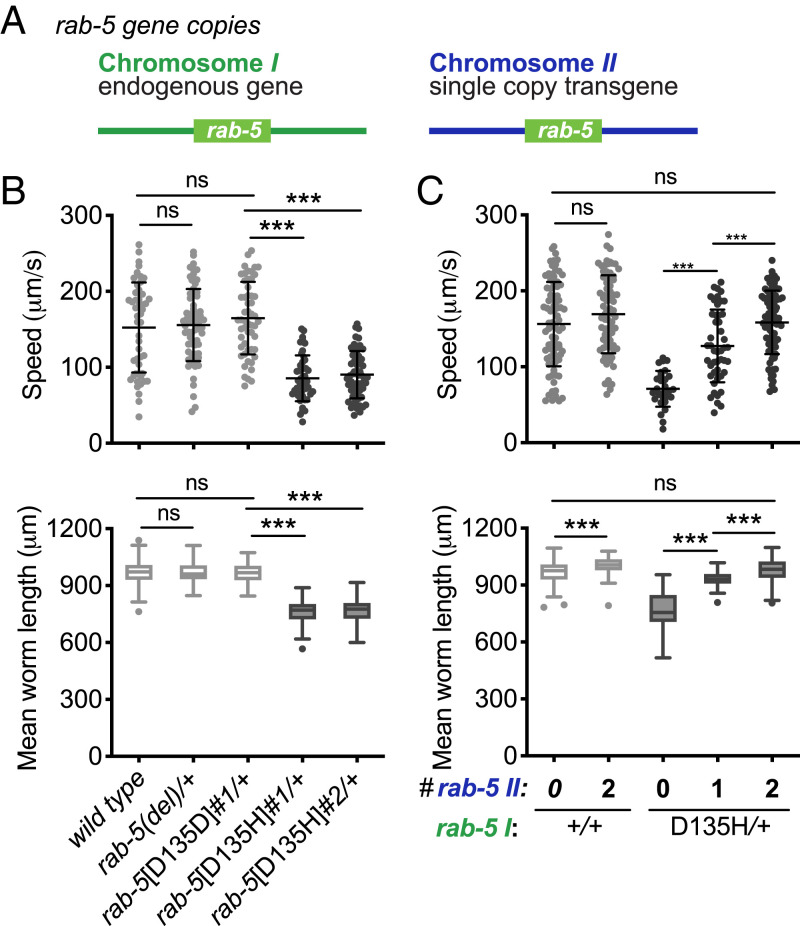
*C. elegans rab-5*[D135H] produces a dominant negative RAB-5 small GTPase. (*A*) Illustration of the endogenous *rab-5* locus on chromosome *I* and the single-copy genomic wild-type *rab-5* transgene integrated into a safe harbor locus on chromosome *II*. (*B*) Mean locomotion speed and worm length on growth media agar plates for the wild-type, *rab-5(del)* heterozygotes, *rab-5*[D135D]*#1* control edit heterozygotes, *rab-5*[D135H]*#1* heterozygotes, and *rab-5*[D135H]*#2* heterozygotes (tested animals were cross-progeny from mating). (*C*) Locomotion speed and mean worm length on growth media agar plates. The wild type and the wild type homozygous for the single wild-type copy insertion of *rab-5* on chromosome *II* (light gray) were self-progeny, while *rab-5*[D135H]*#2* heterozygotes with the normal version of chromosome *II* hemizygous or homozygous for the chromosome *II* containing a single wild-type copy of *rab-5* (dark gray) were cross-progeny from mating. Allele designations for independent edits (#1, #2) are in *SI Appendix*, Table S2. Three independent biological replicates were combined for each genotype. *n* ≥ 46 per condition, except for *rab-5*[D135H]*#2* heterozygotes in *C* (*n* = 29). Scatterplots showing mean and SD are presented for locomotion speed. Filled circles indicate the average speed per animal for up to 1 min. Box plots indicate the mean and first and third quartiles, and whiskers indicate the 5th and 95th percentiles for measures of animal length. Differences between groups were determined by the Student’s *t* test. ns, not significant. ***P < 0.001.

To investigate the functional significance of *RAB5B* p.D136H and the genetic basis of the dominant presentation, we modeled the proband’s variant in *C. elegans*. We generated *rab-5*[D135H] and the *rab-5*[D135D] control animals employing CRISPR-Cas9 editing at the endogenous *rab-5* locus on chromosome *I* (*SI Appendix*, Fig. S3 and Table S2). *rab-5* deletion [*rab-5(del)*] animals were homozygous lethal. Similarly, the *rab-5*[D135H] homozygous animals were lethal, arrested at the first larval stage (L1). In the variant heterozygotes, we quantitatively assessed organismal phenotypes, including locomotion and size (Fig. 2*B*). The *rab-5(del)* and control *rab-5*[D135D] heterozygous animals were normal compared with wild-type animals. However, the *rab-5*[D135H] heterozygotes had a >40% reduction in locomotion and a >20% reduction in size at 24 h post-L4 larval stage. D135H is thus damaging to *rab-5* function and displays a dominant behavior not observed by *rab-5(del)*, consistent with a dominant negative gene product.

By definition, the phenotype of an antimorphic or dominant negative mutation is suppressed by additional copies of the wild-type gene product (37). To test for a dominant negative genetic mechanism of *rab-5*[D135H], we introduced a genomic wild-type *rab-5* single-copy transgene into a chromosome *II* safe harbor site (Fig. 2*A*) and assessed the effect of gene dosage on phenotype. We first confirmed that the wild-type single-copy transgene behaves like the endogenous locus based on the evidence that 1) two copies of *rab-5* transgene fully rescued *rab-5(del)* homozygotes in locomotion (*SI Appendix*, Fig. S3*A*), 2) transcript read counts from the endogenous locus and the transgene were comparable (*SI Appendix*, Fig. S3*B*), and 3) RAB-5 protein level was increased approximately twofold in semiquantitative western blots (*SI Appendix*, Fig. S3 *G* and *H*). We found that animals with wild-type *rab-5* at the endogenous locus and homozygous (two copies) for the chromosome *II rab-5* transgene (i.e., having four copies of wild-type *rab-5*) were slightly longer but normal with respect to locomotion (Fig. 2*C*). For the *rab-5*[D135H] heterozygous animals, one copy of the *rab-5* transgene partially rescued length and locomotion defects, while two copies of the *rab-5* transgene fully rescued the *rab-5*[D135H] heterozygotes in both length and locomotion (Fig. 2*C*). Combined, these results demonstrate that *C. elegans rab-5*[D135H] has a strong dominant negative effect, requiring three copies of the wild-type gene to suppress the variant’s poisonous effect. By extension, we propose that the human *RAB5B* p.D136H is damaging, producing a dominant negative poisonous gene product and resulting in the proband’s phenotype.

### *rab-5*[D135H] Heterozygous Animals Were Defective in Endocytosis and EE Fusion.

**Fig. 3.**
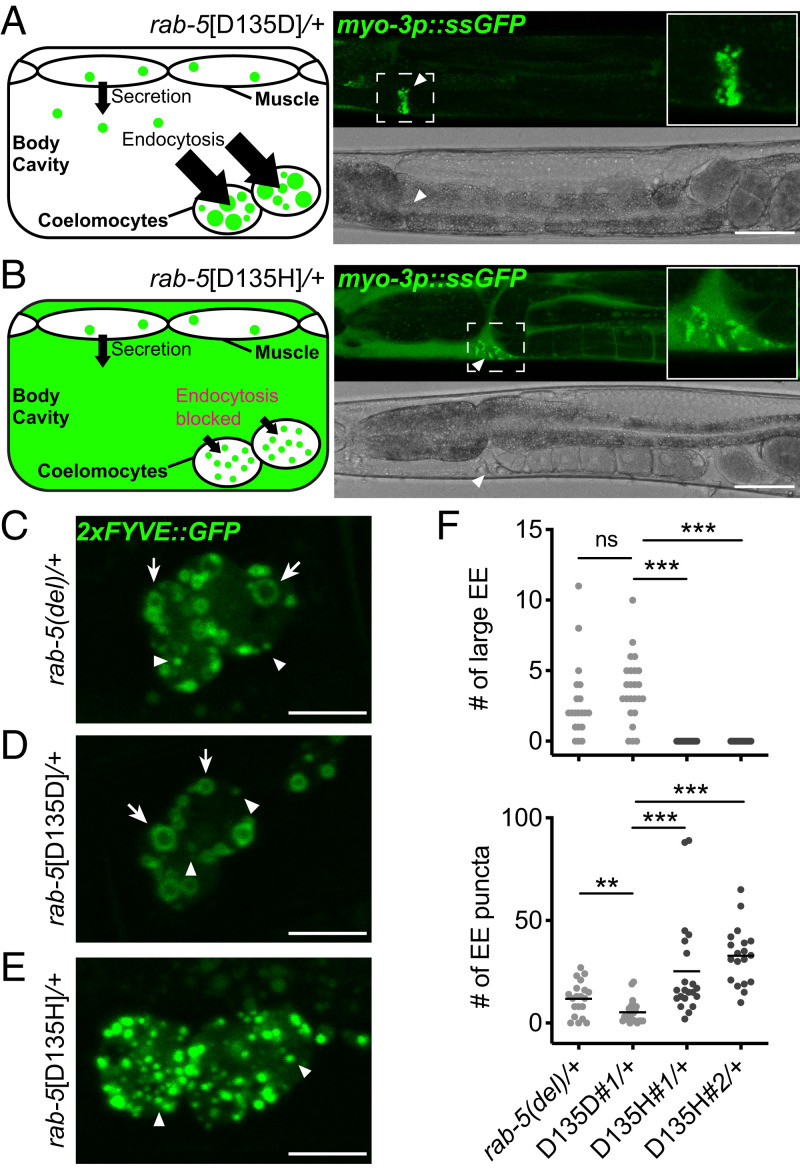
Defective endocytic uptake and fusion in *C. elegans rab-5*[D135H] heterozygotes. (*A*) Steady-state ssGFP level imaged in *rab-5*[D135D]*#1* control edit heterozygous animals 24 h post-L4. Schematic (*Left*) and confocal (*Right*) image showing rapid endocytic uptake of ssGFP from the body cavity into coelomocytes. (*B*) Steady-state ssGFP level imaged in *rab-5*[D135H]*#1* heterozygous animals 24 h post-L4. Schematic (*Left*) and confocal (*Right*) image showing accumulation of ssGFP in the body cavity with limited uptake in coelomocytes. Arrowheads indicate ssGFP in coelomocytes. Quantification of the staining pattern data is in *SI Appendix*, Fig. S4*A*. (Scale bars: 50 μm.) (*C*–*E*) 2xFYVE::GFP-labeled EEs imaged in coelomocytes of *rab-5(del)* heterozygotes (*C*), *rab-5*[D135D]*#1* control edited heterozygotes (*D*), and *rab-5*[D135H]*#1* heterozygotes (*E*). Arrows indicate large ring-shaped EE. Arrowheads indicate small puncta-sized EE. (Scale bars: 10 μm.) (*F*) Quantification of ring-shaped large EE and puncta-sized EE in coelomocytes of *rab-5(del)* heterozygotes, *rab-5*[D135D]*#1* heterozygotes, and *rab-5*[D135H]*#1* and *rab-5*[D135H]*#2* heterozygotes as in *C*–*E*. *n* ≥18 coelomocytes from at least five animals per genotype. Allele designations for independent edits (#1, #2) are in *SI Appendix*, Table S2. Coelomocytes are very large professional cells specialized in endocytic uptake and thus, have large EE, facilitating analysis. ns, not significant. **P < 0.005; ***P < 0.001 determined by the Mann–Whitney *U* test.

During endocytosis in animal cells, RAB5 mediates the heterotypic fusion of nascent endocytic vesicles with EEs and the homotypic fusion of EEs to become large ring-shaped EEs when visualized in cross-section by confocal microscopy. We first assessed the effect of *rab-5*[D135H] on endocytic uptake, measuring fluid-phase endocytosis via clearance of secreted soluble green fluorescent protein (ssGFP) in the body cavity by coelomocytes and receptor-mediated uptake of yolk protein by oocytes (28, 38, 39). *C. elegans* coelomocytes are scavenger cells that nonspecifically endocytose fluid from the body cavity (pseudocoelom) (Fig. 3*A*). Under normal conditions, ssGFP in the body cavity, secreted from muscle cells, is efficiently endocytosed and cleared by the coelomocytes. As expected, in wild-type animals and control *rab-5*[D135D] heterozygotes, the majority of ssGFP was in the coelomocytes (Fig. 3*A* and *SI Appendix*, Fig. S4*A*). In contrast, *rab-5*[D135H] heterozygous animals failed to clear ssGFP from the body cavity, indicating a blockage of endocytic trafficking (Fig. 3*B* and *SI Appendix*, Fig. S4*A*). Similarly, *rab-5*[D135H] heterozygotes failed to accumulate yolk protein, synthesized by the intestine, into oocytes, indicating a blockage of receptor-mediated yolk protein endocytosis (*SI Appendix*, Fig. S4 *B*–*E*). In both assays, *rab-5(del)* heterozygotes were not significantly different from wild-type animals, corroborating the organismal length and locomotion data and indicating that *rab-5* is not haploinsufficient in *C. elegans*.

We next assessed the effect of *rab-5*[D135H] on EE fusion by visualizing the size of EEs in coelomocytes using the marker 2xFYVE::GFP, which binds to phosphatidylinositol-3-phosphate (PI(3)P)-modified lipids on EEs (40, 41). *rab-5(del)* and control *rab-5*[D135D] heterozygous animals contained various stages of EEs, ranging from small-sized puncta to large ring-shaped structures (Fig. 3 *C*, *D*, and *F*), suggesting continuous endosomal trafficking in these animals. In contrast, EEs in *rab-5*[D135H] heterozygotes were all puncta sized and failed to mature into larger ring-shaped EEs (Fig. 3 *E* and *F*), presumably due to the dominant negative RAB-5 D135H disrupting EE fusion. We conclude that *rab-5*[D135H] is defective in EE fusion events, leading to multi-cell type defects in endocytic uptake and EE function. By extension, we suggest that *RAB5B* p.D136H produces a dominant negative RAB5B GTPase that disrupts EE fusion events. However, as humans have three *RAB5* paralogs, there is likely additional biological complexity that arises from potential genetic redundancy and possible paralog-specific RAB5B function.

### Disruption of RAB5B Accumulation in the Proband Lung.

**Fig. 4.**
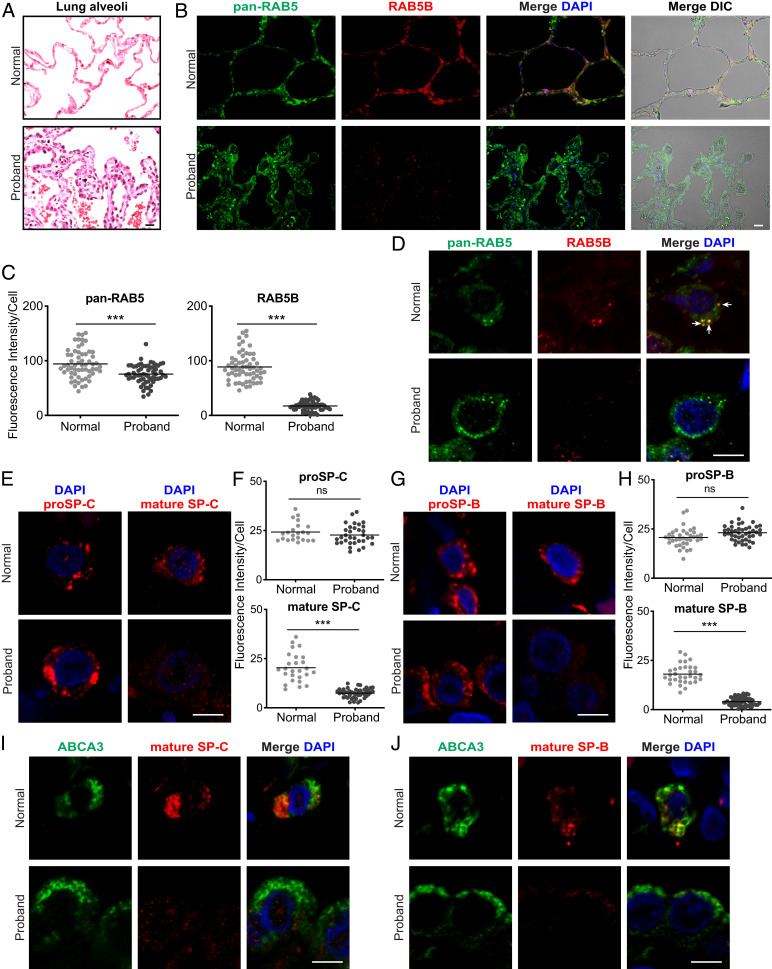
Loss of RAB5B and mature SP-B and SP-C in the proband lung. Lung sections from normal donor and proband lung tissue are stained as indicated. (*A*) AT2 cell hyperplasia in the proband (hematoxylin and eosin). (*B*) Immunostaining for pan-RAB5 and RAB5B (*SI Appendix*, Figs. S13 and S14). Differential interference contrast (DIC) microscopy, together with staining, shows lung structure, with a pentagonal alveolar organization in the normal donor and hyperplastic disorganization in the proband. (*C*) Quantification of immunostaining from *B*. (*D*) Confocal microscopy images of single lung cells with pan-RAB5 and RAB5B antibodies showing colocalization in cytoplasmic puncta (arrows). (*E*) proSP-C and mature SP-C staining and (*G*) proSP-B and mature SP-B staining in single cells. (Scale bars: *A* and *B*, 20 μm; *D, E,* and* G*, 5 μm.) (*F* and *H*) Quantification of immunostaining from low-magnification staining for proSP-C, mature SP-C, proSP-B, and mature SP-B. (*I* and *J*) ABCA3 and mature SP-C staining and ABCA3 and mature SP-B staining, respectively, in individual cells. Images in *A*, *B*, *D*, *E*, *G*, *I*, and *J* are representative of *n* = 3 to 4 normal donor lungs. Nuclei were stained with 4′,6-diamidino-2-phenylindole (DAPI). Shown in *C*, *F*, and *H* are plots of relative fluorescent intensity of cells from *n* = 4 to 6 images from each condition analyzed by the two-tailed Student’s *t* test. ns, not significant. ***P < 0.001.

We next sought to connect the de novo dominant negative *RAB5B* p.D136H allele identified in the proband with ILD and features of a surfactant dysfunction disorder (Fig. 1). From the proband lung biopsy, fixed tissue was available for antibody staining; unfortunately, lung tissue was not available for transmission electron microscopy or western blotting. We first assessed whether *RAB5B* messenger RNA (mRNA) (as well as *RAB5A* and *RAB5C*) was expressed in normal infant lung AT2 cells, the site of surfactant synthesis, from the LungMAP human single-cell RNA sequencing (RNA-seq) dataset (42). The AT2 cell Uniform Manifold Approximation and Projection (UMAP) clusters from day-1 and 21-mo datasets were identified using marker genes *SFTPB* and *SFTPC* (*SI Appendix*, Figs. S5 and S6). For both day 1 and 21 mo, *RAB5B*, *RAB5A,* and *RAB5C* were all expressed in AT2 cells as well as other lung cell types (*SI Appendix*, Fig. S7), with *RAB5A* expression lower than *RAB5B* and *RAB5C*. Detection of *RAB5B* mRNA in these postnatal cells is consistent with the *RAB5B* p.D136H allele affecting AT2 cell function. We propose that RAB5B dysfunction leads to a defect in surfactant protein trafficking/processing, resulting in the child’s ILD (Figs. 1*D* and 4*A*).

As an initial evaluation, we stained normal (transplant donor) and proband lung sections with an antibody that reacts with RAB5A, RAB5B, and RAB5C (pan-RAB5) together with an RAB5B-specific antibody (*SI Appendix*, *SI Materials and Methods* and Fig. S8 have assessment of the specificity of the RAB5 antibodies). The pan-RAB5 antibody stained both normal and proband lung tissue (Fig. 4 *B* and *D*), although immunofluorescent staining was more extensive in the proband due to AT2 cell hyperplasia (AT2 cells were identified by proSP-B, proSP-C, and ABCA3 immunostaining [*SI Appendix*, Figs. S13 and S14]). In contrast, while RAB5B-specific staining was observed in the normal lung, the fluorescent signal was significantly reduced in the proband’s lung tissue. Quantification indicated an ∼80% reduction in RAB5B staining in the proband vs. normal AT2 cells, while pan-RAB5 staining showed an ∼20% reduction in proband vs. normal lung (Fig. 4 *C* and *D*). In contrast, there was no obvious change in RAB5B staining in lung tissues and AT2 cells in patients with surfactant protein dysfunction disorders resulting from pathogenic variants in *SFTPB*, *SFTPC*, or *ABCA3* (*SI Appendix*, Figs. S9–S11 and S14; variants information is in *SI Appendix*, *SI Materials and Methods*), indicating that the reduction in RAB5B staining in the proband is not a consequence of a surfactant dysfunction disorder. The reduction in RAB5B staining in AT2 cells is consistent with the dominant negative *RAB5B* p.D136H allele contributing to the proband’s ILD.

The reduction of RAB5B staining in the proband could be because the dominant negative *RAB5B* variant leads to the degradation of RAB5B or leads to reduced GTP binding, resulting in diffuse cytoplasmic staining. To address this possibility in the absence of a suitable proband lung sample for western blot, we assessed the level of RAB-5 protein in *C. elegans* by western blotting employing an anti–RAB-5 antibody preparation (43). The RAB-5 band was identified by its substantial knockdown following *rab-5* RNA interference (RNAi) feeding and its molecular weight (*SI Appendix*, Fig. S3*C*). We found that the RAB-5 level was significantly reduced in *rab-5*[D135H] heterozygotes compared with control edit *rab-5*[D135D] heterozygotes, with a similar or slightly greater decrease relative to *rab-5(del)* heterozygotes (*SI Appendix*, Fig. S3 *E* and *F*). The decrease in RAB-5 level in variant heterozygotes, in contrast to deletion heterozygotes, is likely a result of protein degradation. Unfortunately, we were unable to evaluate the subcellular distribution of RAB-5 and RAB-5[D135H] as the antibody staining in the intestine of the wild type was not altered following *rab-5* RNAi. This result may be due to cross-reaction with *C. elegans* proteins larger and/or smaller than RAB-5 observed in the western blot (*Materials and Methods* and *SI Appendix*, Fig. S3*C*). The *C. elegans* results with the RAB-5[D135H] heterozygous strains suggest that the reduced RAB5B staining in proband AT2 cells is, at least in part, due to protein degradation.

### Disruption of Mature SP-B and SP-C Production in the Proband Lung.

To more thoroughly investigate and provide insight into the possible disorder of surfactant dysfunction, we immunostained normal and proband lung sections for proSP-B or mature SP-B as well as proSP-C or mature SP-C (Fig. 4 *E*–*H* and *SI Appendix*, Fig. S13; *SI Appendix*, Figs. S9–S12 show assessment of the specificity of the surfactant protein antibodies). proSP-B and proSP-C staining signal intensity per cell was similar in normal and proband lung tissue, with more extensive staining in the proband lung due to AT2 cell hyperplasia. In contrast, the mature SP-B and SP-C staining signal was significantly reduced by ∼66% in the proband vs. normal AT2 cells. The presence of the precursor proteins but not the mature SP-B and SP-C indicates that there is a defect in trafficking/processing in the vesicular pathway for production of mature SP-B and SP-C. These results are consistent with the decreased levels of mature SP-B and SP-C causing a disorder of surfactant dysfunction and ILD in the proband.

ABCA3 is an ABC transporter family member that transports phospholipids into the lamellar body to generate the functional surfactant protein–lipid complex. ABCA3, like proSP-B and proSP-C, is synthesized in the ER and trafficked to the lamellar body, potentially by the same vesicular pathway used for the surfactant protein trafficking. To address this possibility, we stained normal donor and proband lung sections for ABCA3 (Fig. 4 *I* and *J* and *SI Appendix*, Figs. S12, S13, and S15). We observed the previously reported ring-like ABCA3 stained structures (44, 45), with similar intensity and organization, in both normal and proband AT2 cells, while mature SP-B and SP-C staining was significantly reduced only in the proband. The typical staining of ABCA3 in the proband suggests that ABCA3 is trafficked by a different pathway, at least at the level of RAB5, from the surfactant proteins proSP-B and proSP-C. Additionally, the staining showed that lamellar body organization, at least relative to the distribution of ABCA3 immunostaining, was similar in the proband and normal lung.

### proSP-B and proSP-C Colocalize with RAB5B and EEA1 in Normal Donor AT2 Cells.

**Fig. 5.**
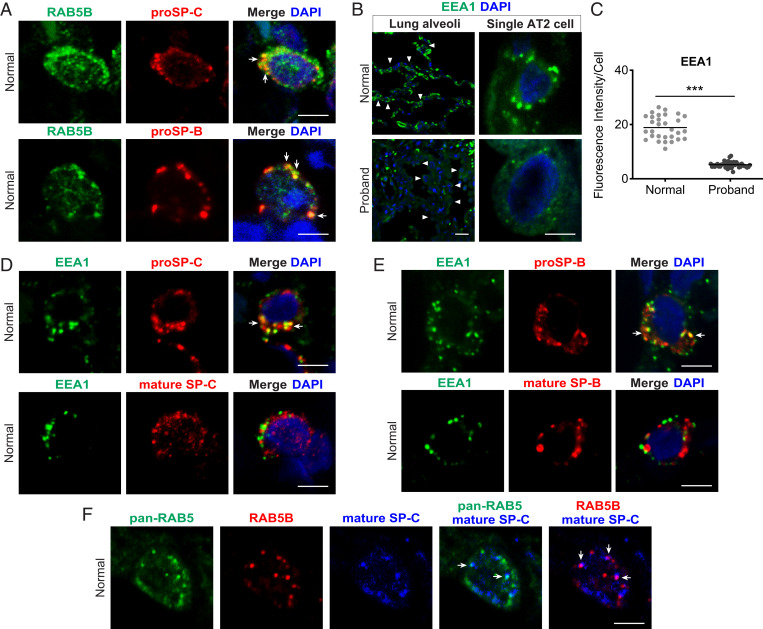
Colocalization of proSP-B and proSP-C with RAB5B and EEA1 in donor AT2 cells. Tissue sections from normal donor lung were immunostained with antibodies for the indicated protein. (*A*) Colocalization of RAB5B and proSP-C or RAB5B and proSP-B (arrows) in single cells. (*B*) EEA1 staining. (*Upper*) Normal donor. (*Lower*) Proband. (*Left*) Low-power image; arrowheads indicate a subset of AT2 cells. (*Right*) Single-cell confocal micrographs are shown. (*C*) Quantification of EEA1 staining from low-power images in *B*. ***P < 0.001, two-tailed Student’s *t* test. (*D* and *E*) Normal donor lung EEA1 and proSP-C or mature SP-C and EEA1 with proSP-B or mature SP-B, respectively, in single cells. (*F*) Pan-RAB5, RAB5B, and mature SP-C in normal lung. Arrows in *A*, *D*, *E*, and *F* indicate colocalization of markers in cytoplasm. Images in *A*, *B*, *D*, *E*, and *F* are representative images of *n* = 3 to 4 normal donor lungs. Nuclei were stained with DAPI. Shown in *C* is the mean fluorescent intensity of immunostained cells from *n* = 4 images for each condition. (Scale bars: *A*, *B*, *Right*, *D*, *E*, and *F*, 5 μm; *B*, *Left*, 20 μm.)

How might RAB5B function in the accumulation of mature SP-B and SP-C while not necessary for the accumulation of proSP-B and proSP-C? RAB5B and EEs have recently been found to have a noncanonical function in the regulated secretion pathways in mammalian mast cells and the *Drosophila* salivary gland (8, 9). RAB5B-associated EEs may similarly fuse with small sorting vesicles containing proSP-B and proSP-C in the surfactant secretion pathway. Alternatively, it is known that extracellular mature SP-B and SP-C are recycled by AT2 cells for repackaging into the lamellar body or trafficked to the lysosomes for degradation (46, 47), presumably through the canonical RAB5B-dependent endocytic pathway. If RAB5B and EEs have a noncanonical function in the regulated secretion pathway for surfactant, we predict that in normal lung, proSP-B and proSP-C would colocalize with RAB5B and EE marker EEA1, and that colocalization with EEA1 would be lost in the proband lung. In contrast, if RAB5B functions only in endocytic recycling/degradation of extracellular mature SP-B and SP-C, colocalization of RAB5B with proSP-B and proSP-C is not expected. Significantly, staining for RAB5B and for proSP-B or proSP-C showed colocalization in normal lung (Fig. 5*A*). Furthermore, proSP-B or proSP-C and EEA1 also showed colocalization in normal lung (Fig. 5 *D* and *E*). While proSP-B and proSP-C staining was similar between normal and proband AT2 cells, EEA1 staining was significantly reduced in the proband lung (Fig. 5 *B* and *C*), precluding assessment of colocalization in proband AT2 cells. Since EE localization of EEA1 is dependent on activated RAB5B, the decreased staining of EEA1 in the proband lung is likely due to the dominant negative *RAB5B* p.D136H. The colocalization of RAB5B with proSP-B and proSP-C, as well as of EEA1 with proSP-B and proSP-C in normal lung, is consistent with RAB5B-dependent fusion of nascent sorting vesicles containing proSP-B and proSP-C with EEs to form small sorting vesicles in the regulated surfactant secretion pathway.

We also examined canonical endocytic recycling of mature SP-B and SP-C. In normal lung, EEA1 colocalized with mature SP-B and SP-C (Fig. 5 *D* and *E*). Mature SP-C staining also showed colocalization with pan-RAB5 and RAB5B (Fig. 5*F*). These results suggest that RAB5B, as well as RAB5A and RAB5C, functions in endocytic recycling of extracellular mature surfactant proteins and is consistent with RAB5B and RAB5C being identified in the lamellar body proteome (48). A function in endocytic recycling of mature SP-B and SP-C (Fig. 5 *D*–*F*) provides an explanation of why some RAB5B did not colocalize with proSP-B and proSP-C (Fig. 5*A*) in the regulated surfactant secretion pathway.

### *Rab5b*-Specific mRNA Knockdown in MLE-15 Cells Results in a Defect in the Formation of Mature SP-B.

**Fig. 6.**
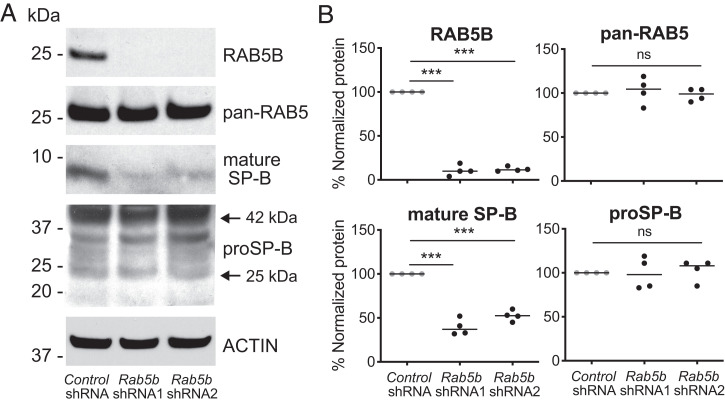
*Rab5b* knockdown in MLE-15 cells decreases mature SP-B production. MEL-15 cells were transduced with lentiviruses that express nontargeted control shRNA or *Rab5b*-specific shRNA sequences (shRNA1, shRNA2) after selection for 5 d in puromycin. (*A*) Western blot analysis of transduced MLE-15 cells using antibodies to detect RAB5B, pan-RAB5 (total RAB5), mature SP-B, proSP-B, and ACTIN. (*B*) Quantification of the western blot signal for RAB5B, total RAB5, mature SP-B, and proSP-B from MLE-15 cells transduced with control shRNA, *Rab5b* shRNA1, and *Rab5b* shRNA2 from four independent transductions. Multiple medians were compared using the Kruskal–Wallis test followed by Dunn’s multiple comparison. ns, not significant. ***P < 0.001.

The above analysis suggests that RAB5B may specifically function in a noncanonical EE fusion event with proSP-B– and proSP-C–containing nascent early sorting vesicles in surfactant trafficking/processing. Accordingly, we performed *Rab5b*-specific knockdown using short hairpin RNAs (shRNAs) in MLE-15 cells. The MLE-15 cell line has AT2 characteristics, was derived from a transgenic mouse with an SV40 transformed lung, and has previously been employed to investigate the processing of proSP-B (49) (*SI Appendix*, Fig. S16). For two different shRNAs, RAB5B protein, but not total RAB5 protein, was significantly knocked down (Fig. 6*A* and *SI Appendix*, Fig. S16). In a western blot assay to distinguish between precursor and processed SP-B, *Rab5b* shRNA knockdown did not result in a significant change in proSP-B compared with control shRNA treatment (Fig. 6). In contrast, *Rab5b* knockdown resulted in a significant reduction in the mature SP-B band (Fig. 6). The RAB5B-specific knockdown in MLE-15 cells recapitulates the failure to generate mature SP-B from proSP-B observed in the *RAB5B* p.D136H proband lung, likely due to an EE vesicular trafficking defect. Together, these results support that *RAB5B* has an essential function in the regulated secretion pathway for lung surfactant.

## Discussion

We present evidence that the de novo heterozygous *RAB5B* p.D136H variant results in a disorder of surfactant dysfunction. The p.D136H variant is not found in gnomAD, is bioinformatically predicted to be damaging, and based on crystal structure, resides in the fourth nucleotide binding domain of Rab/Ras family small GTPases. However, *RAB5B* is not a haplo-insufficient gene. Functional studies with the *C. elegans* ortholog demonstrated that the variant was damaging in vivo, resulting from the production of a strong dominant negative gene product and providing a genetic mechanism for the de novo dominant presentation. Worm studies also showed that the variant was defective in endocytosis, was defective in heterotypic nascent endocytic/sorting vesicle–EE fusion and/or homotypic EE fusion, and led to degradation of the RAB-5 protein. Defects in RAB5B and EEA1 in the proband as a result of the p.D136H variant were supported by histological analysis of the proband’s lung biopsy showing that RAB5B and EEA1 staining was significantly reduced. The reduction in RAB5B was shown not to be an effect of ILD, as it was not observed in other cases with pathogenic variants in known surfactant dysfunction disorder genes. Notably, proband lung staining revealed a disruption in the generation of mature SP-B and SP-C from proSP-B and proSP-C, respectively. Importantly, a requirement for *RAB5B* function in the production of mature SP-B was recapitulated in cell culture. Furthermore, in normal lung, RAB5B and EEA1 colocalized with proSP-B and proSP-C, indicating a role for RAB5B and EEs in normal surfactant biogenesis. This function appears to be unique to *RAB5B*, but not coexpressed paralogs *RAB5A* and *RAB5C*, in AT2 cells. RAB5 paralog–specific functions have been reported for other aspects of biology: for example, epidermal growth factor receptor (EGFR) trafficking (50), RAC GTPase-mediated cell motility (51), and hematopoietic stem and progenitor cell development (52).

The lung phenotype of the proband was less pronounced than that of infants with biallelic loss-of-function variants in *SFTPB* who present with lethal neonatal respiratory distress syndrome and more similar to patients with *SFTPC* dominant variants (10, 19, 20) who demonstrate variable age of ILD onset, severity, and progression. This difference suggests that in the *RAB5B* p.D136H heterozygous proband, there was partial production of mature surfactant protein B through the regulated surfactant secretion pathway, which was sufficient at birth but ultimately, inadequate and led to lung injury in early life. While this work provides evidence for a direct role of the *RAB5B* p.D136H variant in the trafficking/processing of SP-B and SP-C, it does not address the dysmorphic features or neurological phenotype of the proband. However, given the widespread expression including the brain (https://gtexportal.org/home/gene/RAB5B) and the recent finding that *RAB5C* functions in hematopoietic stem and progenitor cell development (52), *RAB5B* may have analogous paralog-specific functions in facial, digit, and/or neural development.

**Fig. 7.**
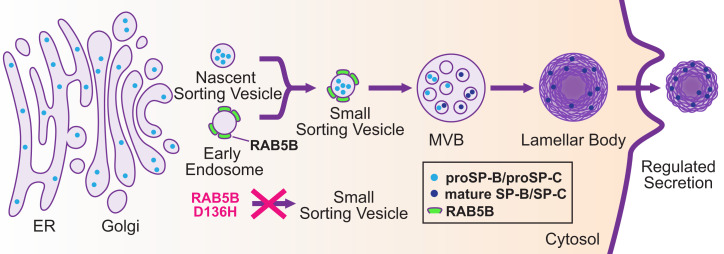
Potential role for RAB5B in the surfactant protein–regulated secretion pathway. A model showing the regulated secretion pathway for surfactant in AT2 cells. Surfactant protein precursors proSP-B and proSP-C are translated in the ER and trafficked to the Golgi, where they bud off as cargo in nascent sorting vesicles. We propose that RAB5B-associated EEs fuse with prosurfactant containing nascent sorting vesicles to form small sorting vesicles, which subsequently fuse with the MVB, and then progress to the lamellar body for maturation and storage. Acidification during vesicular trafficking results in processing of proSP-B and proSP-C to mature SP-B and SP-C in the MVB/lamellar body. We further propose that dominant negative *RAB5B* p.D136H in the proband led to aberrant EEs that were defective in productive fusion with prosurfactant containing nascent sorting vesicles to generate functional small sorting vesicles (indicated by red X). We speculate that aberrant EEs containing RAB5B D136H, potentially as arrested fusion intermediates with nascent sorting vesicles containing proSP-B or proSP-C, are degraded. Such a mechanism would provide an explanation for reduced RAB5B as well as reduced EEA1 and the significant reduction of mature SP-B and SP-C. It is unknown if proSP-B and proSP-C are trafficked in the same or separate vesicles.

The production of mature SP-B and SP-C in AT2 cells by the regulated surfactant secretion pathway has been described (12–16) (Fig. 7). We propose, as a refinement of this pathway, that the nascent sorting vesicles containing proSP-B or proSP-C undergo RAB5B-dependent fusion with EEs, which then mature into MVBs en route to lamellar bodies. This proposal is consistent with colocalization of proSP-B and proSP-C with RAB5B and EEA1, with subsequent acidification during vesicular progression through the regulated secretion pathway resulting in proteolytic processing to form mature SP-B and SP-C and with recent reports of Rab5 and EEs functioning in vesicular progression in mammalian mast cells and the *Drosophila* salivary gland–regulated secretion pathways (8, 9). We further propose that in the proband lung, productive fusion of EEs with the nascent sorting vesicles containing proSP-B or proSP-C is disrupted because of the *RAB5B* p.D136H variant, resulting in a failure to traffic prosurfactant proteins to the MVBs/lamellar bodies (Fig. 7). In contrast, ABCA3 trafficking and formation of the ABCA3 compartment of the lamellar body appear to be normal in the proband. Thus, the failure to produce mature SP-B and SP-C is proposed to be a result of defective vesicular trafficking rather than a failure in protein processing per se. We note that while we observe a defect in the production of both mature SP-B and SP-C in the proband, SP-B function is needed for the generation of mature SP-C (17, 22, 53), so it is possible that only proSP-B trafficking is disrupted by the RAB5B D136H variant protein.

The *C. elegans* experiments revealed that RAB-5 protein is degraded in *rab-5*[D135H] heterozygous animals. However, the gene dosage studies demonstrated that it is not the reduced levels of RAB-5 causing the heterozygous mutant phenotypes but the dominant negative poisonous D135H gene product, which requires three wild-type *rab-5* copies to fully restore normal phenotypes. Instead, the degradation of RAB-5 is likely a secondary effect of the dominant negative protein. By extension, the significantly reduced RAB5B level in proband AT2 cells is likely due to protein degradation, a secondary effect of the dominant negative *RAB5B* p.D136H gene product. RAB5B D136H could be removed by a ubiquitin-mediated degradation process. Alternatively, aberrant EEs containing RAB5B D136H, possibly engaged in defective fusion with proSP-B or proSP-C containing nascent sorting vesicles, could be recognized as abnormal and removed through autophagy (54). This latter possibility is intriguing as it could also explain both the reduced levels of EEA1 and the ultimate fate of the surfactant proteins as well as why levels of proSP-B and proSP-C did not build up in proband AT2 cells when vesicular trafficking was disrupted.

A limitation of this work is that proband lung tissue samples for transmission electron microscopy to assess vesicular and lamellar body ultrastructure and for western blotting to assess processing of the endogenous surfactant proteins were not available. Mouse knock-in models of *Rab5B* p.D136H as well as *Rab5B* AT2 cell conditional knockout should allow testing of the model (Fig. 7) and assessment of whether the other phenotypes in the proband (e.g., neurological phenotypes) are also caused by the dominant negative *RAB5B* variant.

In summary, we have used modeling in *C. elegans*, knockdown studies in MLE-15 cells, and staining of proband lung tissue to provide evidence that the de novo heterozygous *RAB5B* p.D136H allele is a genetic mechanism causing surfactant dysfunction that was previously unknown. Currently, this is a single case, however, it is likely other *RAB5B* variants, especially dominant negative variants or compound heterozygous variants, may cause disease. We suggest that for infants and children with ILD consistent with a disorder of surfactant dysfunction in whom a pathogenic variant in the established surfactant disease genes is not found, the *RAB5B* locus be sequenced.

## Materials and Methods

The *SI Appendix* has more details.

### Human Studies.

Written consent for genetic studies, for use of tissue for research, and to publish photographs was obtained from the proband’s parents. Genetic and tissue studies were approved by the institutional review boards (IRBs) at Washington University in St. Louis and Baylor College of Medicine. The UDN protocol, 15-HG-0130, was approved by the National Human Genome Research Institute IRB. Nondiseased control lungs were those donated but unsuitable for transplantation. Lung sections from individuals with disorders of surfactant dysfunction due to pathogenic variants in *SFTPB*, *SFTPC*, and *ABCA3* were used for comparisons with the immunostaining from the proband. Parents provided written consent, and the study was approved by the IRB at Washington University School of Medicine in St. Louis.

### *C. elegans* Culture and Strain Information.

*C. elegans* strains were cultured on standard nematode growth media seeded with OP50 *Escherichia coli* and grown at 20 °C (55). *rab-5*[D135H] heterozygous animals showed significant cold sensitivity, although they can be very slowly propagated at 15 °C; we, therefore, maintained all *rab-5*[D135H] and corresponding control strains at 22 °C and assayed them at 20 °C. Strains and alleles used in this study are listed in *SI Appendix*, Table S2.

### *C. elegans* CRISPR-Cas9 Gene Editing.

Single-nucleotide changes were introduced by editing the VC2010 animals with CRISPR-Cas9 (56, 57) (*SI Appendix*, Table S3). A single-copy *rab-5* transgene was integrated at Mos1 transposon insertion site *ttTi5605* on chromosome *II* (*II*: 0.77cM) (58) through CRISPR-Cas9 using the self-excising cassette method (59). All CRISPR-edited strains were backcrossed at least twice.

### WormLab Assays and Analyses.

Crawling assays were performed using the WormLab system (MBF Bioscience) for individual worm tracking and analysis. Age/stage-matched young adults were analyzed for locomotion and size (60). Assays for endocytosis were performed by crossing into various marker strains and tested in cross-progeny. Images were analyzed using LASX (Leica Microsystems) and Volocity (v6.3; Quorum Technologies).

### Immunostaining and Image Analysis.

Tissue sections were processed and immunostained using primary antibodies as in *SI Appendix*, Table S4. Images for lung sections were acquired using an epifluorescent or laser scanning confocal microscope and were then analyzed for fluorescent intensity in FIJI (61).

### *Rab5b* RNAi.

Control nontargeted (SHC002; Sigma-Aldrich) and mouse *Rab5b*-specific shRNAs (Sigma-Aldrich) (*SI Appendix*, Table S5) were used to produce lentivirus and transduce MLE-15 cells similar to the methods previously described (62).

### Immunoblot Analysis.

Proteins from MLE-15 cells were separated onto 4 to 12% gradient polyacrylamide gels. Membranes were incubated with the primary and secondary antibodies in *SI Appendix*, Table S4. The density of bands was quantified relative to ACTIN in FIJI.

### Statistics.

Statistical analysis was performed using GraphPad Prism (version 6.07). Differences between two groups were compared using the two-tailed Student’s *t* test or the Mann–Whitney *U* test. Multiple medians were compared using the Kruskal–Wallis test followed by Dunn’s multiple comparison. *P* < 0.01 indicates statistically significant difference, unless otherwise stated. Box plots show the median, a box representing the first and third quartiles, and whiskers at the 5th and 95th percentiles.

## Supplementary Material

Supplementary File

## Data Availability

All study data are included in the article and/or *SI Appendix*.
