# Supplementary material for: A dominant negative variant of RAB5B disrupts maturation of surfactant protein B and surfactant protein C
Source: Proc Natl Acad Sci U S A. 2022 Feb 4;119(6):e2105228119. doi: 10.1073/pnas.2105228119 (PMC8832968; doi:10.1073/pnas.2105228119)
Supplement: Supplementary File [file pnas.2105228119.sapp.pdf]

Supplementary Information for

A dominant negative variant of *RAB5B* disrupts maturation of surfactant protein B and surfactant protein C

Huiyan Huang[a,1], Jiehong Pan[b,1], David R. Spielberg[c], Neil Hanchard[d], Daryl A. Scott[d,e], Lindsay C. Burrage[d], Hongzheng Dai[d], David Murdock[d], Jill A. Rosenfeld[d], Ariz Mohammad[f], Tao Huang[b], Anika G. Lindsey[a], Hyori Kim[a], Jian Chen[f], Avinash Ramu[f], Stephanie A. Morrison[a], Zachary D. Dawson[a], Alex Z. Hu[a], Eric Tycksen[g], Gary A. Silverman[a], Dustin Baldridge[a,f], Jennifer A. Wambach[a], Undiagnosed Diseases Network[2], Stephen C. Pak[a,*], Steven L. Brody[b,*], Tim Schedl[f,*]

a. Department of Pediatrics, Washington University School of Medicine, St. Louis, MO 63110
b. Department of Medicine, Washington University School of Medicine, St. Louis, MO 63110
c. Department of Pediatrics, Section of Pulmonary Medicine, Baylor College of Medicine, Houston TX 77030
d. Department of Molecular and Human Genetics, Baylor College of Medicine, Houston, TX 77030
e. Department of Molecular Physiology and Biophysics, Baylor College of Medicine, Houston, TX 77030
f. Department of Genetics, Washington University School of Medicine, St. Louis, MO 63110
g. Genome Technology Access Center, McDonnell Genome Institute, Washington University School of Medicine, St. Louis, MO 63110

[1] H.H. and J.P. contributed equally to this work.
[2] A complete list of the Undiagnosed Diseases Network consortium is followed below.
[*] To whom correspondence may be addressed. Email: ts@wustl.edu (primary), brodys@wustl.edu, Stephen.pak@wustl.edu

This file includes:

SI Materials and Methods
Tables S1 to S5
Figures S1 to S16
SI References

CONSORTIA (Members of the Undiagnosed Diseases Network)

Maria T. Acosta, Margaret Adam, David R. Adams, Justin Alvey, Laura Amendola, Ashley Andrews, Euan A. Ashley, Mahshid S. Azamian, Carlos A. Bacino, Guney Bademci, Ashok Balasubramanyam, Dustin Baldridge, Jim Bale, Michael Bamshad, Deborah Barbouth, Pinar Bayrak-Toydemir, Anita Beck, Alan H. Beggs, Edward Behrens, Gill Bejerano, Hugo Bellen, Jimmy Bennet, Beverly Berg-Rood, Jonathan A. Bernstein, Gerard T. Berry, Anna Bican, Stephanie Bivona, Elizabeth Blue, John Bohnsack, Devon Bonner, Lorenzo Botto, Brenna Boyd, Lauren C. Briere, Elly Brokamp, Gabrielle Brown, Elizabeth A. Burke, Lindsay C. Burrage, Manish J. Butte, Peter Byers, William E. Byrd, John Carey, Olveen Carrasquillo, Thomas Cassini, Ta Chen Peter Chang, Sirisak Chanprasert, Hsiao-Tuan Chao, Gary D. Clark, Terra R. Coakley, Laurel A. Cobban, Joy D. Cogan, Matthew Coggins, F. Sessions Cole, Heather A. Colley, Cynthia M. Cooper, Heidi Cope, William J. Craigen, Andrew B. Crouse, Michael Cunningham, Precilla D'Souza, Hongzheng Dai, Surendra Dasari, Joie Davis, Jyoti G. Dayal, Matthew Deardorff, Esteban C. Dell'Angelica, Katrina Dipple, Daniel Doherty, Naghmeh Dorrani, Argenia L. Doss, Emilie D. Douine, Laura Duncan, Dawn Earl, David J. Eckstein, Lisa T. Emrick, Christine M. Eng, Cecilia Esteves, Marni Falk, Liliana Fernandez, Elizabeth L. Fieg, Laurie C. Findley, Paul G. Fisher, Brent L. Fogel, Irman Forghani, William A. Gahl, Ian Glass, Bernadette Gochuico, Rena A. Godfrey, Katie Golden-Grant, Madison P. Goldrich, Alana Grajewski, Irma Gutierrez, Don Hadley, Sihoun Hahn, Rizwan Hamid, Kelly Hassey, Nichole Hayes, Frances High, Anne Hing, Fuki M. Hisama, Ingrid A. Holm, Jason Hom, Martha Horike-Pyne, Alden Huang, Yong Huang, Wendy Introne, Rosario Isasi, Kosuke Izumi, Fariha Jamal, Gail P. Jarvik, Jeffrey Jarvik, Suman Jayadev, Orpa Jean-Marie, Vaidehi Jobanputra, Lefkothea Karaviti, Jennifer Kennedy, Shamika Ketkar, Dana Kiley, Gonench Kilich, Shilpa N. Kobren, Isaac S. Kohane, Jennefer N. Kohler, Deborah Krakow, Donna M. Krasnewich, Elijah Kravets, Susan Korrick, Mary Koziura, Seema R. Lalani, Byron Lam, Christina Lam, Grace L. LaMoure, Brendan C. Lanpher, Ian R. Lanza, Kimberly LeBlanc, Brendan H. Lee, Hane Lee, Roy Levitt, Richard A. Lewis, Pengfei Liu, Xue Zhong Liu, Nicola Longo, Sandra K. Loo, Joseph Loscalzo,

Richard L. Maas, Ellen F. Macnamara, Calum A. MacRae, Valerie V. Maduro, Rachel Mahoney, Bryan C. Mak, May Christine V. Malicdan, Laura A. Mamounas, Teri A. Manolio, Rong Mao, Kenneth Maravilla, Ronit Marom, Gabor Marth, Beth A. Martin, Martin G. Martin, Julian A. Martínez-Agosto, Shruti Marwaha, Jacob McCauley, Allyn McConkie-Rosell, Alexa T. McCray, Elisabeth McGee, Heather Mefford, J. Lawrence Merritt, Matthew Might, Ghayda Mirzaa, Eva Morava, Paolo M. Moretti, John J. Mulvihill, Mariko Nakano-Okuno, Stan F. Nelson, John H. Newman, Sarah K. Nicholas, Deborah Nickerson, Shirley Nieves-Rodriguez, Donna Novacic, Devin Oglesbee, James P. Orengo, Laura Pace, Stephen C. Pak, J. Carl Pallais, Christina GS. Palmer, Jeanette C. Papp, Neil H. Parker, John A. Phillips III, Jennifer E. Posey, Lorraine Potocki, Barbara N. Pusey, Aaron Quinlan, Wendy Raskind, Archana N. Raja, Deepak A. Rao, Anna Raper, Genecee Renteria, Chloe M. Reuter, Lynette Rives, Amy K. Robertson, Lance H. Rodan, Jill A. Rosenfeld, Natalie Rosenwasser, Francis Rossignol, Maura Ruzhnikov, Ralph Sacco, Jacinda B. Sampson, Mario Saporta, C. Ron Scott, Judy Schaechter, Timothy Schedl, Kelly Schoch, Daryl A. Scott, Vandana Shashi, Jimann Shin, Rebecca Signer, Edwin K. Silverman, Janet S. Sinsheimer, Kathy Sisco, Edward C. Smith, Kevin S. Smith, Emily Solem, Lilianna Solnica-Krezel, Ben Solomon, Rebecca C. Spillmann, Joan M. Stoler, Jennifer A. Sullivan, Kathleen Sullivan, Angela Sun, Shirley Sutton, David A. Sweetser, Virginia Sybert, Holly K. Tabor, Amelia L. M. Tan, Queenie K.-G. Tan, Mustafa Tekin, Fred Telischi, Willa Thorson, Cynthia J. Tifft, Camilo Toro, Alyssa A. Tran, Brianna M. Tucker, Tiina K. Urv, Adeline Vanderver, Matt Velinder, Dave Viskochil, Tiphanie P. Vogel, Colleen E. Wahl, Stephanie Wallace, Nicole M. Walley, Melissa Walker, Jennifer Wambach, Jijun Wan, Lee-kai Wang, Michael F. Wangler, Patricia A. Ward, Daniel Wegner, Monika Weisz-Hubshman, Mark Wener, Tara Wenger, Katherine Wesseling Perry, Monte Westerfield, Matthew T. Wheeler, Jordan Whitlock, Lynne A. Wolfe, Jeremy D. Woods, Kim Worley, Changrui Xiao, Shinya Yamamoto, John Yang, Diane B. Zastrow, Zhe Zhang, Chunli Zhao, Stephan Zuchner

SI Materials and Methods

Exome sequencing and Variant analysis.
DNA, from the proband and parents, was extracted from peripheral blood mononuclear cells for trio exome sequencing (ES) in CLIA certified laboratories and variants were confirmed by Sanger sequencing. Trio ES was performed at Baylor Genetics using methods described (1). Produced sequence reads were aligned to the GRCh37 (hg19) human genome reference assembly then variants were determined and called using Edico Dragen BioIT Platform to generate a variant call file. The annotation platform leverages the GenomOncology Knowledge Management System API and provides annotations using open source data sets such as gnomAD, EVS, and ClinVar, and professional resources such as HGMD Pro. Baylor Genetics provided a clinical analysis of the proband's trio ES.

For the research analysis of the trio exome data, Codified Genomics (www.codifiedgenomics.com) was used to prioritize novel, de novo variants that were not observed in gnomAD and biallelic variants in the coding region or near intron-exon boundaries with an allele frequency of less than 1% (2) (**Table S1**). In addition, copy number variant (CNV) analysis was performed using the XHMM tool (3) that compares depth-of-coverage in exome sequencing data. XHMM was run on >200 exomes sequenced at Baylor Genetics using the same library and sequencing parameters to generate baseline coverage statistics and identify outliers for CNV discovery. The list of genotyped CNVs was then annotated for genes, gnomAD frequency, ClinVar, and other relevant annotations using AnnotSV (4). Custom scripts were then used to identify rare and de novo CNVs for additional investigation.

Phylogenetic tree generation and multi-sequence alignment. The phylogenetic tree was generated with Clustal Omega (https://www.ebi.ac.uk/Tools/msa/clustalo/). Protein sequences of H.s. RAB5A (UniProt #P20339), H.s. RAB5B (UniProt #P61020), H.s. RAB5C (UniProt #P51148), D.m. Rab5 (UniProt #Q9V3I2), and C.e. RAB-5 (UniProt #P91857) were downloaded from UniProt.

Multiple sequence alignment was performed using T-Coffee (http://tcoffee.crg.cat/apps/tcoffee/do:regular). The "fasta_aln" result was then shaded with BoxShade (https://embnet.vital-it.ch/software/BOX_form.html).

***C. elegans* CRISPR-Cas9 gene editing.** Single nucleotide changes were introduced by injecting VC2010 animals with Cas9 protein, tracrRNA, crRNA, and single-stranded DNA oligonucleotide (ssODN) repair template (5, 6). The repair templates were designed to have greater than 33-bp homology on each arm and include synonymous changes that destroy gRNA re-binding and create or destroy a restriction site for genotyping. Lines with the synonymous changes, but not the proband variant, were generated as controls to verify that the silent

mutations did not contribute to the observed phenotype (referred to as control edits). The *dpy-10* co-conversion strategy (7) was employed to enrich for edited events. Edited alleles were considered independent when obtained from different injected animals. The *rab-5* gene in the edited strains was Sanger sequenced to verify that no extraneous changes in the gene were introduced. Strains were backcrossed twice to remove non-linked background mutations. We retained and analyzed two independent control edits and three independent variant edits to assess possible phenotypes from unrelated variations that might have arisen from off-target editing or segregation in VC2010. Preliminary experiments indicated that control edit strains D135D #1 and #2 were phenotypically similar and not different from VC2010; we, therefore, used #1 in all subsequent experiments. Similarly, preliminary experiments indicated that variant edited strains D135H #1, #2, and #3 were phenotypically similar; we therefore used #1 and #2 in subsequent experiments. See **Table S2** for the complete strain genotype.

A single copy *rab-5* transgene was integrated at the safe harbor Mos1 transposon insertion site *ttTi5605* on Chromosome *II* (*II*: 0.77cM) (8) through CRISPR-Cas9. The single copy transgene allele was generated via the Self Excising Cassette (SEC) method (9). The SEC contains the *sqt-1* roller marker and hygromycin antibiotic selection marker to facilitate screening (9). The small guide RNA (sgRNA seq: *atatcagtctgtttcgtaa*) plasmid for *ttTi5605* MOS-SCI site was cloned into plasmid DR274 U6 through *Bsa*I site. The *rab-5* transgene contains the genomic DNA sequence from 1.3 kb upstream of the *rab-5* start codon to 0.6 kb downstream of the stop codon (chromosome I: 9,307,157 to 9,310,370). Two synonymous changes were introduced at amino acid position 84 (TTG to TTA) and 133 (AAG to AAA) to facilitate the comparison of mRNA level between the *rab-5* wild type and transgene locus in RNA-seq analysis.

***C. elegans* locomotion and length analysis.** Worm length and crawling speed were measured using the WormLab system (MBF Bioscience), a video recording instrument fitted with software for tracking and analyzing individual worms (10). Age/stage matched young adults were used. Briefly, 35 late L4 stage larvae per strain were picked onto a thin-lawn assay plate 24 hours before the assay. On the day of assay, 10 animals were transferred to each assay plate, allowed to recover for 20 minutes, then video recorded for 2 minutes. Three plates per strain were recorded per trial and three independent trials were performed for each experiment, totaling up to 90 animals assessed per strain. Within each trial, plates were scrambled to avoid systematic effects. Videos were analyzed using WormLab software. When multiple tracks were generated from one worm, tracks were manually joined whenever possible. If a worm moved out of and re-entered the field, tracks were not manually joined and the longer tracking data was used. Any worms that tracked less than 15 seconds were censored from analysis. A custom R script was written to compile the data files and aggregate the worm length (mean worm length) and speed (center point speed) data (see GitHub repository: https://github.com/samorrison19/WormLabAnalysis). Two-tailed

Student's t-test was used for statistical analysis and we consider p values less than 0.01 to be significant.

Obtaining animals of the appropriate genotype by crosses for phenotyping. For heterozygous animals tested in WormLab, where there is no visible marker to differentiate self and cross progeny, animals were raised on *fem-1* RNAi plates and females were picked to cross with corresponding males. Strains for endocytosis analysis, specifically *arIs37[myo-3p::ssGFP]*, *pwIs23[vit-2::GFP]*, and *cdIs85[pcc1::2xFYVE::GFP]*, were crossed with VC2010 males to obtain heterozygous marker males, which were then crossed into control or *rab-5*[D135H]*/tmC18* females. The resulting cross progeny that lost the balancer fluorophore but obtained the marker signal were picked at L4 stage and imaged 24 hours later.

Analysis of *rab-5* endogenous and transgene transcript levels by RNA-seq. RNA-seq analysis was conducted in triplicate to compare the mRNA level/read counts between the endogenous *rab-5* wild type locus (Chromosome *I*) and single copy transgene locus on Chromosome *II*, using the strain UDN100087. In brief, 1-day old young adults were collected to isolate total RNA with Trizol reagent following the manufacturer's instruction. Total RNA was further purified with DNase digestion followed by column extraction. Libraries for Next Generation Sequencing were prepared using the manufacturer's recommended protocol by the Genome Technology Access Center. Library fragments were sequenced on an Illumina NovaSeq-6000 using paired-end reads extending 150 bases. The resulting reads were then quasi-aligned and quantitated against the Ensembl WBcel235.90 transcriptome with copies of the wild type and transgenic *rab-5* using the expectation-maximization algorithm in Salmon (11) to generate allele-specific expression in transcripts per million (TPM). The results indicate that the mRNA level of the single copy *rab-5* transgene is not significantly different from the endogenous locus (**Fig. S3**), and thus can be considered as supplying an equivalent gene dose as the wild type *rab-5* gene.

***C. elegans* western blot.** For analysis of *C. elegans* proteins, 100 1-day old young adults were picked into RIPA lysis buffer (G-Biosciences) supplemented with Pierce™ Halt™ protease inhibitor cocktail (Thermo Fisher Scientific) and went through two cycles of freeze-thaw with liquid N_2. Samples were then placed into 2x Laemmli Sample Buffer (Bio-Rad) supplemented with 2-mercaptoethanol (MP Biomedicals) and boiled for 10 min. Samples were centrifuged at 500 x *g* for 2 min and the supernatant separated by 12% Mini-PROTEAN® TGX Stain-Free™ precast gels (Bio-Rad) under reducing conditions, electrotransferred to a polyvinylidene difluoride (PVDF) membrane via Trans-Blot® Turbo transfer system (Bio-RAD) and blocked in Intercept® blocking buffer (LI-COR) for 1 hour at room temperature. Proteins were detected using an anti-RAB-5 rabbit polyclonal antibody (1:100 dilution, gift from Anne Spang) (12) and anti-UNC-15 monoclonal antibody (1:500 dilution, DSHB) overnight at 4°C. The primary antibody binding was detected with anti-rabbit IRDye 800CW (LI-COR) and anti-

mouse StarBright B700 (Bio-RAD) secondary antibodies. Signal was detected using Image LabTM (Bio-RAD). Western blot quantification was performed in ImageJ with the built-in Analyze Gels function. There was considerable variability between western blots, leading to normalization with the relevant control strain depending on the comparison to be made (**Figure S3D, F, H**).

***rab-5* RNA interference.** *rab-5* feeding RNAi bacteria from the Ahringer library (13) was employed to test the specificity of the anti-RAB-5 antibody for both western blotting and cytological staining. Ten gravid adults were transferred to *rab-5* RNAi plates and progeny grown to adults after 3 or 4 days were picked for analysis.

Cytological staining of *C. elegans* intestinal and gonadal preparations. Immunostaining was performed essentially as previously described (14). Anti-RAB-5 antibody staining in the wild type intestine, at the level of the lumen, revealed puncta of various sizes as well as diffuse cytoplasmic staining. Following feeding wild type animals with *rab-5* RNAi bacteria, intestines showed equivalent cytological staining compared to untreated animals. In contrast, western blots showed a significant reduction in the accumulation of the ~23kD band in similarly RNAi-treated animals (**Fig S3**). These results indicate that the cytological staining pattern observed was not RAB-5-specific, likely arising from cross reaction with proteins larger or smaller than RAB-5 observed in the western blot.

Infant normal lung scRNA-seq. Single-cell RNA-seq data was downloaded from the LungMAP consortium portal on April 7, 2020 (15) for analysis by Seurat version 3.1.5. We visualized the cells in 2-dimensions using the UMAP algorithm and identified clusters in an unsupervised manner using the Louvain algorithm (16). From the day-1 and 21-month normal lung datasets, the type II pneumocyte UMAP cluster was identified from reads of marker genes *SFTPB* and *SFTPC* (17). We assessed gene expression differences between the *RAB5A*, *RAB5B*, and *RAB5C* genes using the Wilcoxon Rank Sum test in R.

Immunostaining human lung sections. Formalin fixed tissue sections on glass slides were rehydrated for staining with hematoxylin and eosin or antibodies as indicated (**Table S4**). For immunochemistry, tissue sections were heated in antigen unmasking solution pH 6.0 (Vector Laboratories) in a pressure cooker (Biocare Medical) for 5 min then cooled for 15 min. Tissues were then incubated in tissue blocking buffer (fish gel, 2%, Sigma-Aldrich; donkey serum 5%, Sigma Aldrich; Triton X-100, 0.2% in PBS) for 40 min at room temperature (RT). Primary antibodies diluted in blocking buffer were applied to samples overnight at 4°C. After washing with Tween-20 (0.1%) in PBS, samples were incubated with secondary antibodies for 30 min at RT. Tissues were washed and counterstained with 4', 6 diamidino-2-phenylindole (DAPI) in mounting media (Fluoroshield with DAPI, Sigma) prior to adding the coverslip. Lung sections from individuals with

disorders of surfactant dysfunction: an infant with homozygous *SFTPB* null variants: p.Pro133Glufs*95 (variant previously known as '121ins2') ('*SFTPB null')* (18), an infant compound heterozygous for *ABCA3* null variants: c.817_821del/c.1729_1730del ('*ABCA3 null')*, and an adolescent heterozygous for *SFTPC* p.Ile73Thr ('*SFTPC* missense variant') (19) were used for comparisons to the immunostaining from the proband (de novo *RAB5B* p.Asp136His variant).

***Rab5b* RNAi.** Mouse *Rab5b*-specific shRNA sequences (**Table S5**) in lentivirus vectors selected from the RNAi Consortium library (https://www.broadinstitute.org/rnai-consortium/rnai-consortium-shrna-library) were purchased from Sigma-Aldrich. Lentivirus was produced in HEK293T cells by cotransfection with plasmids VSV-G pMD2.G (Addgene #12259) and psPAX2 (Addgene #12259 and #12260, both a gift of Didier Trono) as described (20). Lentivirus was concentrated using the Lenti-X system (Takara Bio). MLE-15 cells on Transwell membranes (Corning) were transduced with different concentrations of lentivirus in protamine (10 μg/mL) overnight, then two days later selected in puromycin (5 μg/mL) for 3-5 days prior to harvest for analysis.

Immunoblot analysis of *Rab5b* RNAi in MLE-15 cells. For validation of RAB5 proteins, purified proteins purchased from ProSpec (RAB5B: PRO-056, RAB5A: PRO-590, RAB5C: PRO-1427) were separated on a 4-12% Bis-Tris gel, transferred by capillary diffusion to PVDF membrane. Membranes were blocked in blotto (5% dried milk in TBS/0.2% Tween), incubated with anti-goat RAB5 (1:500, Biocompare orb153348) or anti-mouse RAB5B (1:200, Santa Cruz Biotechnology) antibody overnight at 4°C, then species-specific horseradish peroxidase labeled secondary antibodies for detection on X-ray film using chemiluminescence (ECL, Thermo Scientific 32106). For detection of SP-B in MLE-15 cells, following shRNA transfection, cells were lysed in RIPA lysis buffer and proteins (50 μg) were separated on 4-12% Bis-Tris gel and processed for RAB5 and RAB5B antibodies as above, and anti-rabbit proSP-B (1:1000, Millipore AB3430) anti-rabbit mature SP-B (1:1500, Seven Hills WRAB-48604) antibodies.

Image acquisition and analysis. For the ssGFP and 2xFYVE::GFP imaging, animals were placed in 5 µl of 100 mM NaN_3 (Millipore-Sigma) in PBS, in a 35 mm cover glass bottom dish (MatTek). Approximately 15 to 20 adult stage animals were transferred to the NaN_3 solution and covered with a 12-mm circular coverslip and then a 25-mm square coverslip. Confocal images were taken with a Lecia SP8X tandem scanning confocal microscope with a white light laser using either a 40x 1.3 NA oil PlanApo objective over ≥20 z-planes and a pinhole size of 1.00 (Leica Microsystems). Images were displayed as maximum intensity projections. Images were rendered and analyzed using LASX (Leica Microsystems) and Volocity (v6.3; Quorum Technologies, CAN) software.

For the VIT-2::GFP imaging, animals were anesthetized with levamisole, transferred to an agar pad formed on a slide, imaged with a Zeiss compound microscope, and analyzed with Axiovision.

For lung sections, images were acquired using an epifluorescence microscope interfaced with imaging software (LAS X, Leica) or laser scanning confocal microscope (LSM 710; Carl Zeiss, Oberkochen). Images were globally adjusted for brightness and contrast in Photoshop (Adobe). Fluorescence intensity per cell in lung tissues was quantified using the Analyze function for Set Measurement of Integrated density in FIJI (21).

Table S1. Trio exome sequencing analysis

Gene Name	Genomic Coordinates	Transcript	Variant	gnomAD	Inheritance	Notes
RAB5B	12:56384556 G>C	NM_002868.3	c.406G>C, p.(Asp136His)	Not present	de novo[1]	CADD =29.6
AKR1C3	10:5144398 C>T	NM_003739.5	c.676C>T, p.(Arg226*)	Not present	de novo	Exon 6 of 9; pLI=0
CD48	1:160651313 G>C	NM_001778.2	c.386-55C>G	Not present	de novo	CADD =1.5
DDR2	1:162745627 C>A	NM_001014796.1	c.2042C>A, p.(Thr681Asn)	Not present	de novo	CADD =23.1 pLI=0.55
CASKIN1	16:2231447 G>A	NM_020764.3	c.1922C>T, p.(Pro641Leu)	101/272004 (no homozygotes)	Inherited from father	CADD =21.7
CASKIN1	16:2228928 G>C	NM_020764.3	c.4174, p.(Arg1392Gly)	Not present	Inherited from mother	CADD =15.9
GPRIN3	4:90170203 A>C	NM_198281.2	c.1059T>G, p.(Phe353Leu)	96/251028	Inherited from mother	CADD <1
GPRIN3	4:90170324 G>A	NM_198281.2	c.938C>T, p.(Pro313Leu)	96/250922	Inherited from mother	CADD =16.51
GPRIN3	4:90169863 C>T	NM_198281.2	c.1399G>A, p.(Ala467Thr)	Not present	Inherited from father	CADD =1.88
MMACHC[2]	1:45974628 A>G	NM_015506.2	c.590A>G, p.(Asn197Ser)	1/249556	Inherited from father	CADD =25.2
MMACHC[2]	1:45974800 ACCCGCC>A	NM_015506.2	c.766_771delGCCCCC, p.(Ala256_Pro257del)	121/277934	Inherited from mother	
MUC16	19:9083247 C>A	NM_024690.2	c.8568G>T, p.(Glu2856Asp)	4/246764	Inherited from father	CADD <1
MUC16	19:9086805 ACTT>A	NM_024690.2	c.5007_5009delAAG, p.(Arg1669del)	5/280490	Inherited from mother	
OPLAH	8:145113980 G>A	NM_017570.3:	c.366C>T, p.(Ala122=)[3]	14/189938	Inherited from father	CADD =5.34
OPLAH	8:145109945 G>A	NM_017570.3	c.2396C>T, p.(Thr799Met)	15/181224	Inherited from mother	CADD =29.2
SHROOM1	5:132161642 C>G	NM_001172700.1	c.191G>C, p.(Arg64Pro)	Not present	de novo (unknown if variant is on maternal or paternal allele)	CADD =24.5
SHROOM1	5:132161124 C>T	NM_001172700.1	c.709G>A, p.(Gly237Arg)	Not present	Inherited from mother	CADD =21.5
TTN	2:179578857 G>A	NM_001267550.1	c.26528C>T, p.(Thr8843Met)	105/279952	Inherited from father	CADD =22.9
TTN	2:179404451 A>G	NM_001267550.1	c.98341T>C, p.(Cys32781Arg)	62/248458	Inherited from mother	CADD =23.8

Variants identified by research analysis of the trio exome sequencing. de novo variants that are not observed in gnomAD and biallelic variants in the coding region or near intron-exon boundaries with an allele frequency of less than 1% are listed above.

[1]Variant observed in one read in the father (1/141) but not in mother (0/125).

[2]Normal homocysteine indicated that a diagnosis of cobalamin C deficiency was unlikely.

[3]Synonymous change.

Table S2. *C. elegans* strains

Strain Name	Genotype	Description and references
VC2010	Wild-type	Wild-type (22)
UDN100020	*rab-5(ok2605)/tmC18[dpy-5(tmIs1200[myo-2p::Venus])] I*	*rab-5* deletion allele (23). Balancer marked with *myo-2p::Venus* (24).
UDN100022	*rab-5(udn11)/tmC18[dpy-5(tmIs1200[myo-2p::Venus])] I*	*rab-5*[D135D]#*1*. This study.
UDN100021	*rab-5(udn10)/tmC18[dpy-5(tmIs1200[myo-2p::Venus])] I*	*rab-5*[D135D]#2. This study.
UDN100026	*rab-5(udn12)/tmC18[dpy-5(tmIs1200[myo-2p::Venus])] I*	*rab-5*[D135H]#*1*. This study.
UDN100028	*rab-5(udn14)/tmC18[dpy-5(tmIs1200[myo-2p::Venus])] I*	*rab-5*[D135H]#2. This study.
UDN100027	*rab-5(udn13)/tmC18[dpy-5(tmIs1200[myo-2p::Venus])] I*	*rab-5*[D135H]#*3*. This study.
UDN100087	*udnSi38[rab-5p::rab-5] II*	single copy of the wild type *rab-5* locus integrated into chromosome *II*. This study.
UDN100059	*rab-5(ok2605) I; udnSi38[rab-5p::rab-5] II*	*rab-5* deletion with single copy of wild type *rab-5* integrated into chromosome *II*. This study.
UDN1000138	*rab-5(udn11); udnSi38[rab-5p::rab-5] II*	*rab-5* control edit #1 with single copy of wild type *rab-5* integrated into chromosome *II*. This study.
UDN100067	*rab-5(udn14); udnSi38[rab-5p::rab-5] II*	*rab-5* variant edit #2 with single copy of wild type *rab-5* integrated into chromosome *II*. This study.
GS1912	*arIs37[myo-3p::ssGFP + dpy-20(+)] I; dpy-20(e1282) IV*	integrated array with ssGFP expressed in muscle cells (25)
NP941	*unc-119(ed3) III; cdIs85[pcc1::2xFYVE::GFP + myo-2p::GFP + unc-119(+)]*	integrated array with early endosome marker 2xFYVE expressed in coelomocytes (26)
RT130	*pwIs23[vit-2::GFP]*	integrated array with yolk protein VIT-2 fused with GFP expressed in intestinal cells (26)

Table S3. ***rab-5*** **endogenous locus editing details**

gRNA	GATGGCATTGGCTGGAAACA
D135H repair template	AGGCATCTCCAAATATTGTGATGGCATTGGCTGGAAACAAAGCACATGTTGCCAATAAGCGAACTGTTGAGTATGAAGAAGCTAATG
D135D repair template	AGGCATCTCCAAATATTGTGATGGCATTGGCTGGAAACAAAGCAGATGTTGCCAATAAGCGAACTGTTGAGTATGAAGAAGCTAATG
Genotyping primer forward	TTCAGGTGTGCCTTGACGATG
Genotyping primer reverse	TCGTTCACGTTCATTGAGGTCTG

Table S4. Antibodies used

Antibody	Host	Dilution	Company	Catalog #
Primary				
RAB5	Goat	1:200 IF<br>1:500 WB	Biocompare	orb153348
RAB5B	Mouse	1:100 IF<br>1:200 WB	Santa Cruz Biotechnology	sc-373725
proSP-C	Rabbit	1:500 IF	Millipore	AB3786
mature SP-C	Rabbit	1:1000 IF	Seven Hills	WRAB-MSPC
proSP-B	Rabbit	1:1000 IF and WB	Millipore	AB3430
mature SP-B	Rabbit	1: 1000 IF<br>1:1500 WB	Seven Hills	WRAB-48604
EEA1	Mouse	1:300 IF	BD Biosciences	610456
ABCA3	Mouse	1:1000 IF	Seven Hills	WMAB-ABCA3-17
Actin	Mouse	1:2000 WB	Millipore	Mab1501R
RAB-5 (C.e.)	Rabbit	1:100 WB<br>1:1000 IF	Gift from Anne Spang	
UNC-15 (C.e.)	Mouse	1:500 WB	DSHB	5-23
Secondary				
Anti-Goat Alexa 488	Donkey	1:1000	ThermoFisher	A-11055
Anti-Mouse Alexa 555	Donkey	1:1000	ThermoFisher	A-21424
Anti-Rabbit Alexa 555	Donkey	1:1000	ThermoFisher	A-31572
Anti-Mouse Alexa 555	Donkey	1:1000	ThermoFisher	A-21424
Anti-Rabbit Alexa 647	Donkey	1:1000	ThermoFisher	A-31573
Anti-rabbit IRDye 800CW	Goat	1:5000	LI-COR	926-32211
Anti-mouse StarBright B700	Goat	1:5000	Bio-RAD	12004158
Anti-mouse IgG, HRP	Goat	1:5000	Invitrogen	31430
Anti-Rabbit IgG, HRP	Goat	1:5000	Invitrogen	31460
Anti-Goat IgG, HRP	Bovine	1:5000	Jackson Immuno Research	805-035-180

Table S5. *Rab5b* shRNA sequences

**shRNAs**	**Target sequence**
shRNA1	AGCCAGCCCTAGCATTGTTAT
shRNA2 (in **Fig. S16**)	TCGGGCAAAGACATGGGTAAA
shRNA3	CAAAGGACAGTTCCATGAATA
shRNA4	GGAAGTCTAGCCTGGTGTTAC
shRNA5 (renamed as shRNA2 in **Fig. 6**)	TATGAAGAGGCTCAGGCATAT

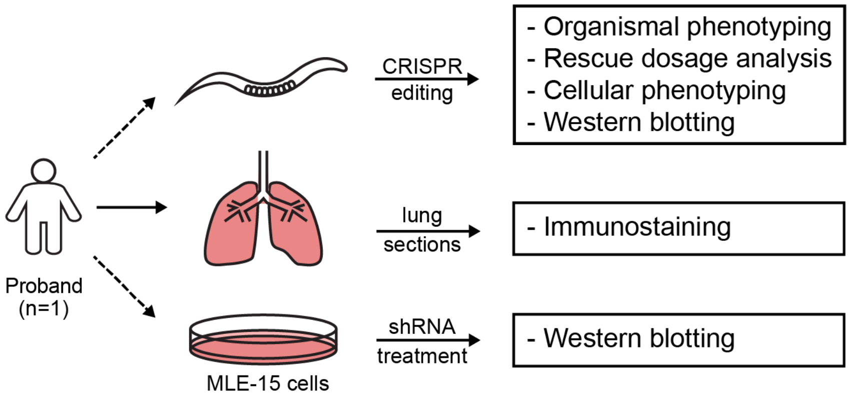

Fig. S1. Outline of functional studies. Schematic indicating the functional studies performed in *C. elegans*, staining in proband and normal lung biopsy sections, and *Rab5b* mRNA specific knockdown in mouse AT2 cell-like SV-40 transformed cell line MLE-15.

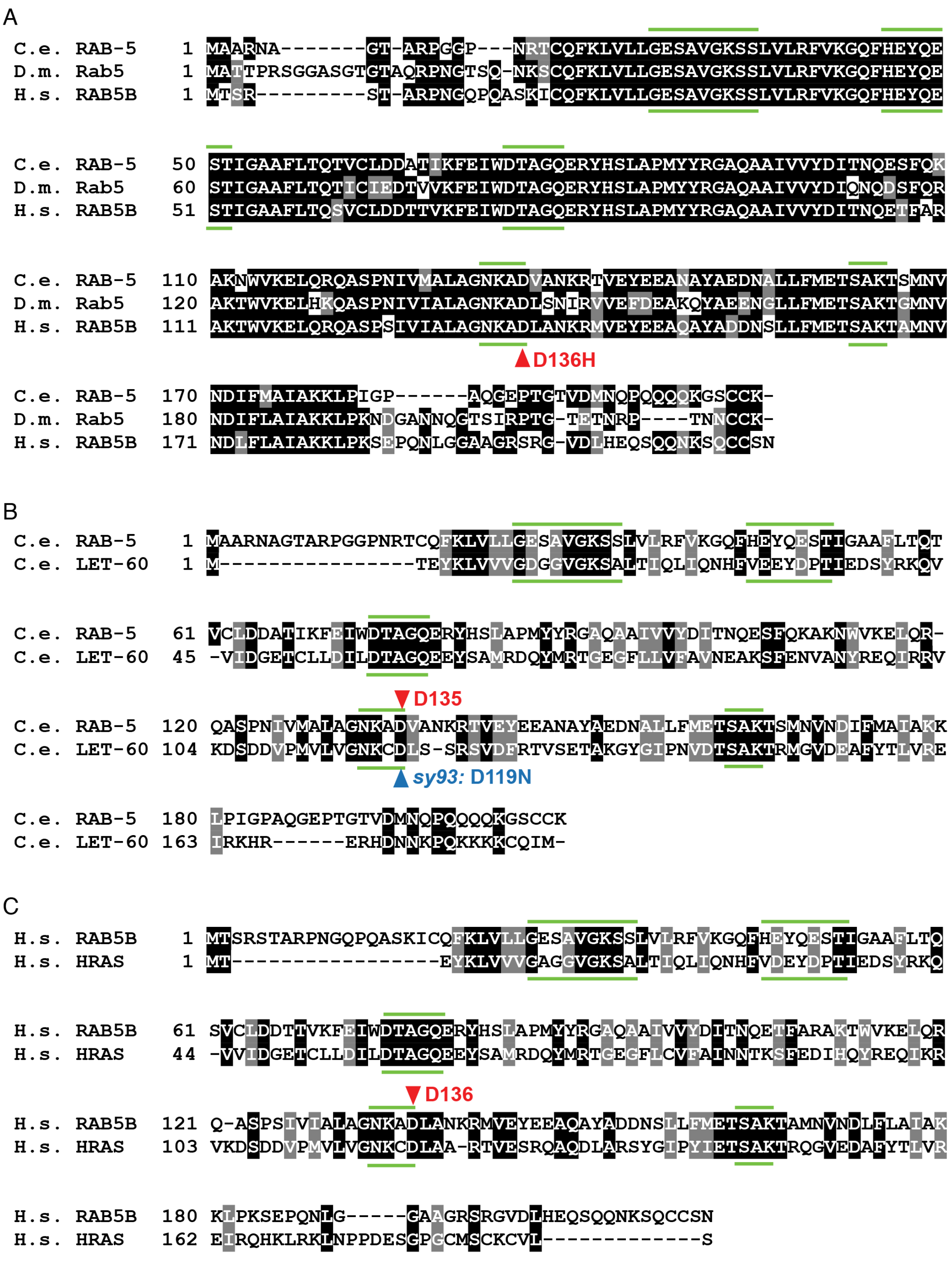

Fig. S2. The RAB5B variant D136 is conserved among Rab5 from different species and with HRAS. (A) Alignment of human (H.s.) RAB5B full-length amino acid sequence with *Drosophila* (D.m.) and *C. elegans* (C.e.) orthologs. The location of the variant in the conserved aspartate [D] in the proband is

indicated (red arrowhead). (B) Alignment of *C. elegans* RAB-5 and LET-60, the *C. elegans* HRAS ortholog. The corresponding proband variant in RAB-5 is indicated (red arrowhead). The dominant negative variant *let-60(sa93)* is indicated (blue arrowhead). (C) Alignment of human RAB5B and HRAS. The location of the variant in the conserved aspartate [D] in the proband is indicated (red arrowhead). The five conserved regions of the nucleotide binding domain are indicated (green bars above and below each of the alignments). Identical residues are shaded black; conserved residues are grey.

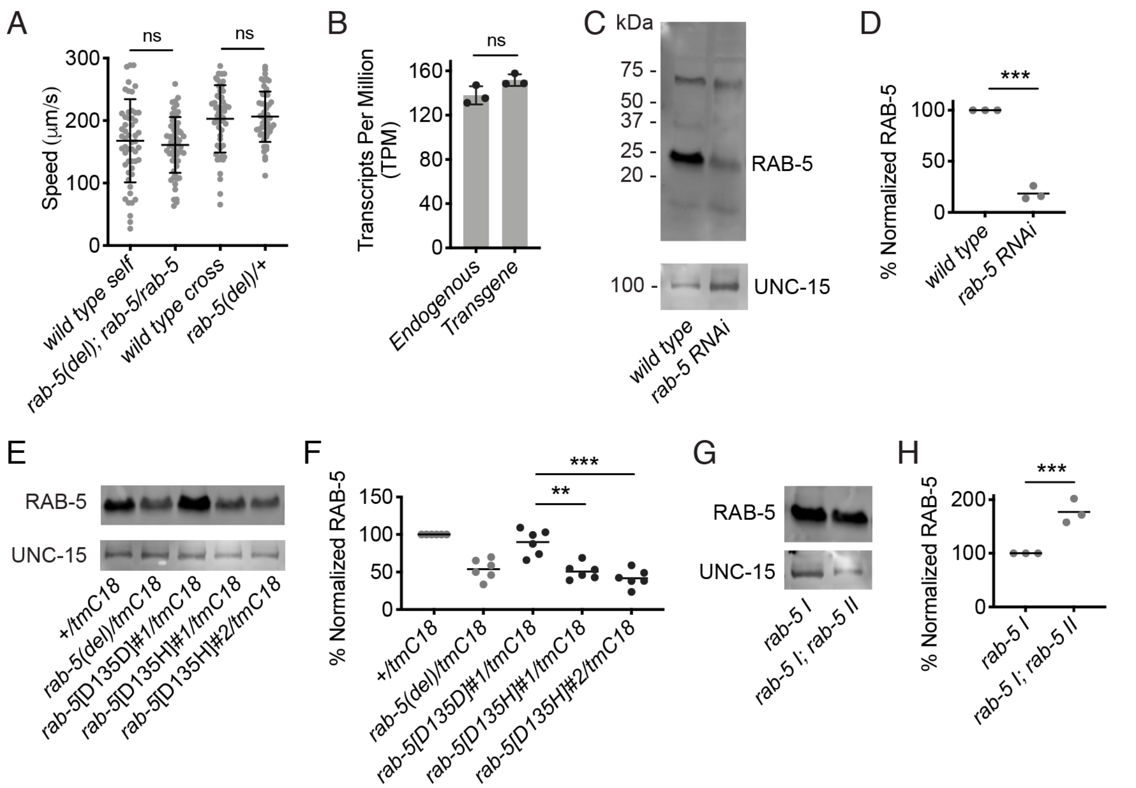

Fig. S3. Analysis of the genomic wild type *rab-5* transgene and RAB-5 protein levels in edited strains. (A) Locomotion speed on NGM agar plates for wild type, hermaphrodites homozygous for *rab-5(del)* and the wild type single copy *rab-5* transgene on chromosome II (both self-progeny), wild type, and *rab-5(del)* heterozygotes (both cross progeny). Scatter plots with mean and standard deviation are shown for locomotion speed. Three independent biological replicates were combined for each genotype. N was at least 50 animals per genotype. Each filled circle indicates the average speed of one animal for up to 1 minute. ns: not significant. (B) RNA-seq transcripts per million (TPM) for the endogenous wild type *rab-5* locus on chromosome *I* and for the wild type *rab-5* transgene on chromosome *II*, distinguished by two synonymous sequence changes in the *rab-5* transgene. RNA was isolated from UDN100087. The bars show the average TPM value from three biological replicates. Filled circles represent individual data points from three biological replicates. The Mann-Whitney U test was used to compare TPM of the *rab-5* endogenous and transgene locus. ns, not significant. (C), (E) and (H) Western blots of 100 animals (except that 50 animals were used for wild type in C and *rab-5 I; rab-5 II* in G) of the indicated genotypes for either RAB-5 (above) or UNC-15 (below). (C) shows the full gel while (E) and (H) only show the RAB-5 region of the gel at ~23kD. (D), (F) and (H) Quantification of RAB-5 staining, relative to the UNC-15 loading control, normalized to the comparator strain, whose value was set to 100%. **, $p<0.005$ and ***, $p<0.001$, by Student's t-test.

A

Genotype	# of animals with ssGFP mainly in coelomocytes	# of animals with ssGFP mainly in the body cavity
+/ssGFP	35	0
rab-5(del)/ssGFP	15	0
rab-5[D135D]*#1/ssGFP*	16	0
rab-5[D135H]*#1/ssGFP*	0	25
rab-5[D135H]*#2/ssGFP*	0	14

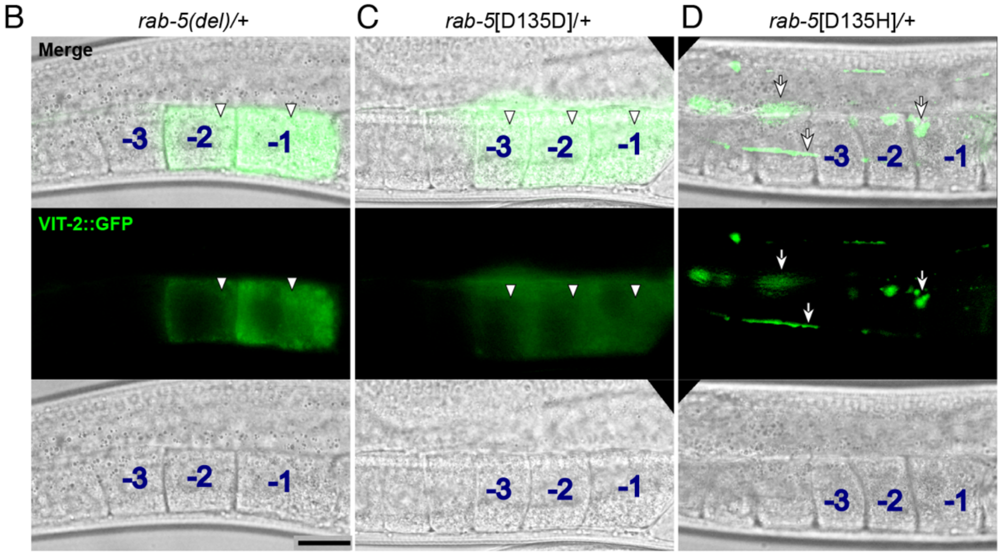

E

Genotype	# of gonad arms with VIT-2::GFP in oocyte	# of animals with VIT-2::GFP in the body cavity
rab-5(del)/+; vit-2::gfp/+	19	0
rab-5[D135D]*#1/+; vit-2::gfp/+*	20	1
rab-5[D135H]*#1/+; vit-2::gfp/+*	0	21

Fig. S4. ssGFP uptake quantification and VIT-2::GPF yolk protein uptake and storage. (A) Quantification of ssGFP endocytosis. (B-D) VIT-2::GFP accumulation in oocytes of *rab-5(del)* heterozygotes (B), *rab-5*[D135D] control edited heterozygotes (C), and *rab-5*[D135H] variant heterozygotes (D). Arrowheads, VIT-2::GFP endocytosed into oocytes; arrows, VIT-2::GFP in the body cavity. VIT-2::GFP is endocytosed into the most proximal oocytes, -1 and -2 (upper right in each panel), prior to their ovulation. In (C), auto-fluorescence in the middle and lower left is from the intestine. In (D), VIT-2::GFP fluorescence signal is outside the oocytes/germline, either between the gonad arms or between the gonad and body wall muscle. Scale bar: 25 μm. (E) Quantification of VIT-2::GFP accumulation in oocytes or in the body cavity.

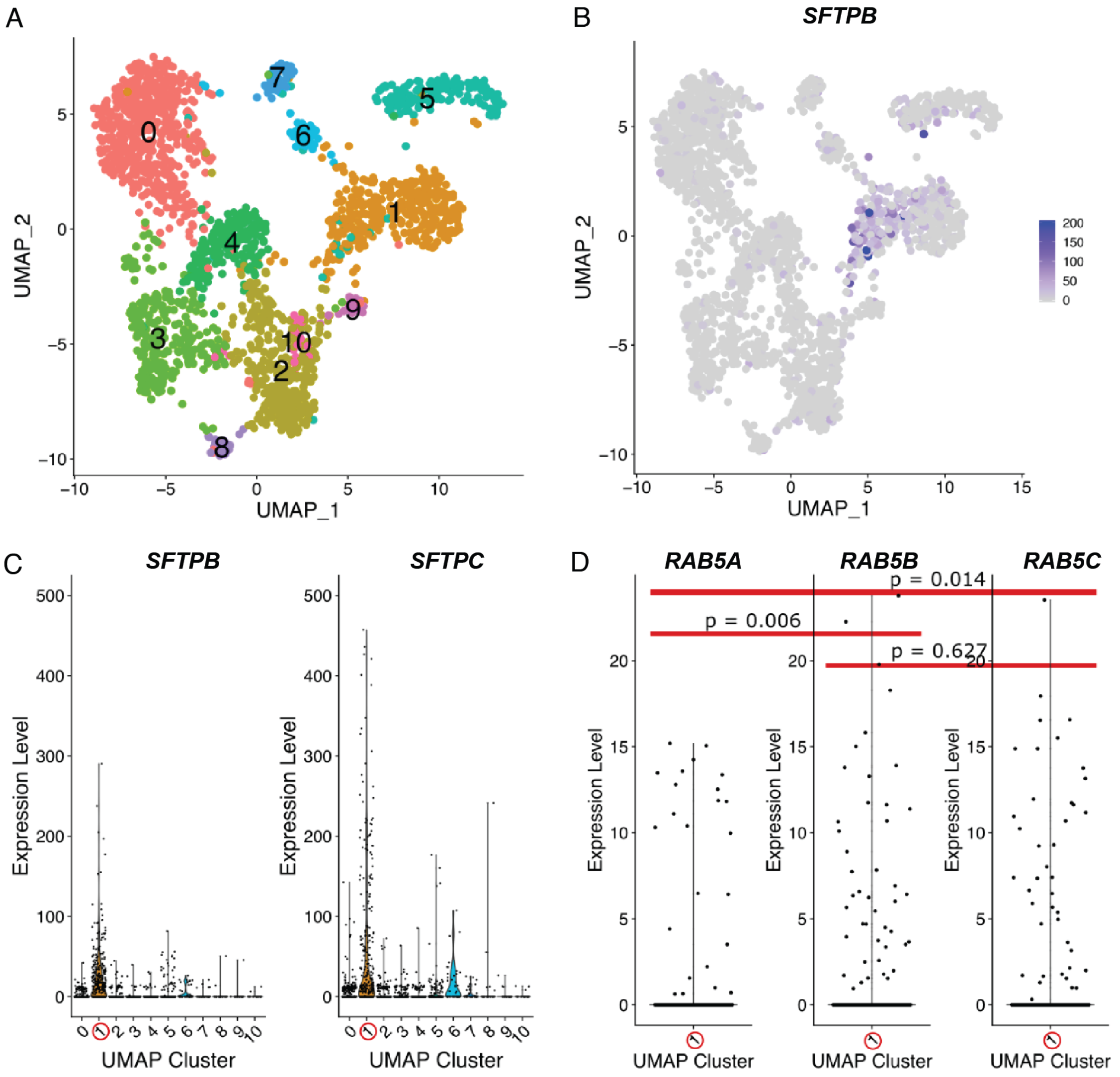

Fig. S5. Gene expression in single-cells from day-1 human lung identifies *RAB5* gene expression pattern in AT2 cells. (A) UMAP clustering of single-cell RNA-seq data from LungMAP. We identified eleven clusters using unsupervised clustering. These clusters reflect putative cell types in the 1-day lung. (B) Cluster 1 marks putative AT2 cells based on the expression of marker gene *SFTPB*. (C) AT2 cell marker genes *SFTPB* and *SFTPC* are both expressed at significantly higher levels in cluster 1 (red circles) compared to other clusters. (D) Expression of *RAB5A*, *RAB5B,* and *RAB5C* in putative AT2 cells from cluster 1 (red circles). *p*-values shown were computed using the Wilcoxon Rank Sum Test. *RAB5A* is expressed at a lower level than both *RAB5B* and *RAB5C*. *RAB5B* and *RAB5C* are not significantly different in their expression levels.

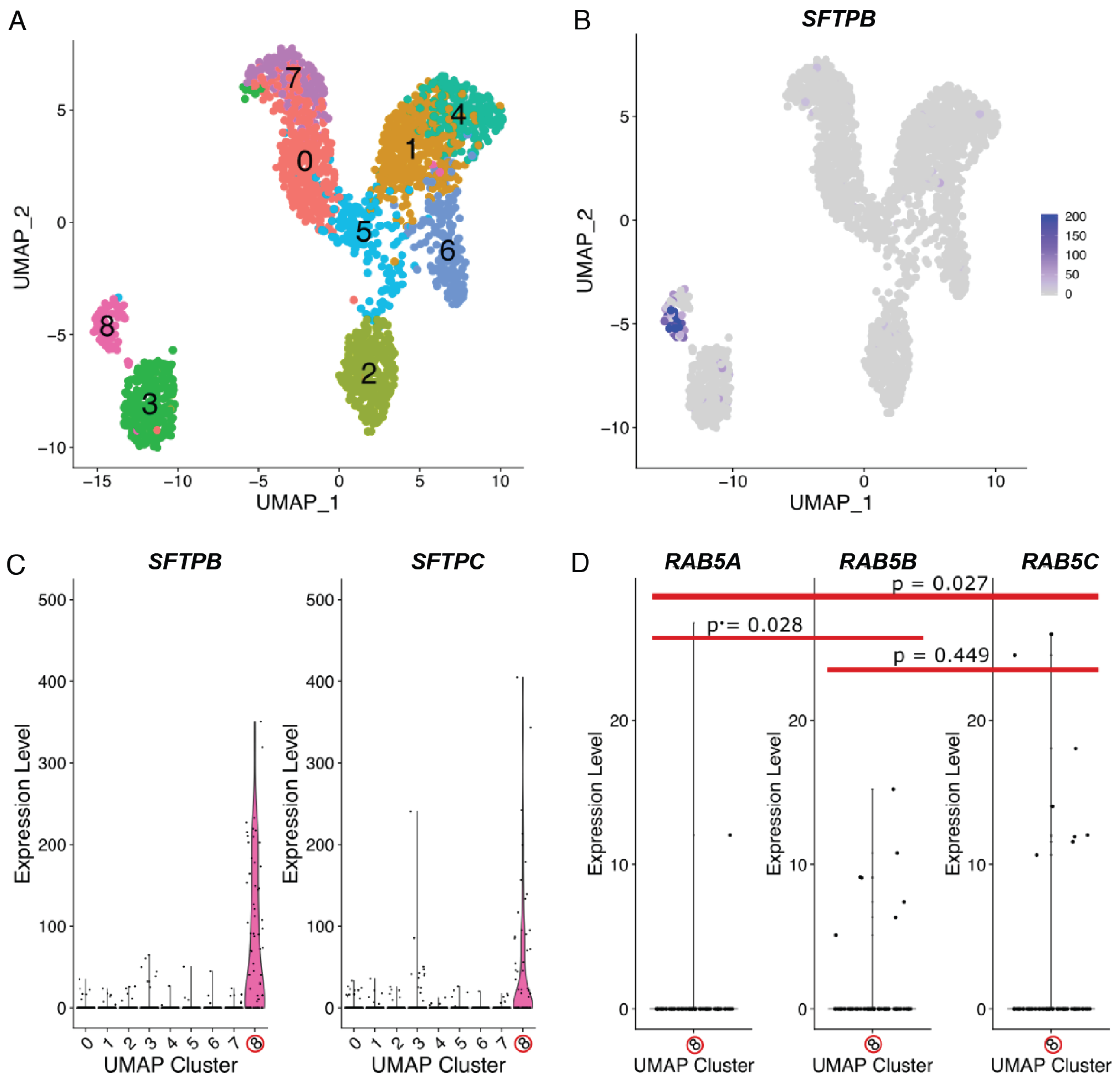

Fig. S6. Gene expression in single cells from 21-month human lung identifies *RAB5* gene expression pattern in AT2 cells. (A) UMAP clustering of single-cell RNA-seq data from LungMAP. Nine clusters were identified using unsupervised clustering. Clusters reflect putative cell types in the 21-month lung. (B) Cluster 8 marks putative AT2 cells based on expression of marker gene *SFTPB*. (C) AT2 cell marker genes *SFTPB* and *SFTPC* are expressed at significantly higher levels in cells belonging to cluster 8 (red circles) compared to other clusters of cells. (D) Expression of *RAB5A*, *RAB5B*, and *RAB5C* in putative AT2 cells from cluster 8 (red circles). *p*-values shown were computed using the Wilcoxon Rank Sum Test. *RAB5A* is expressed at a lower level than both *RAB5B* and *RAB5C*. *RAB5B* and *RAB5C* are not significantly different in their expression levels.

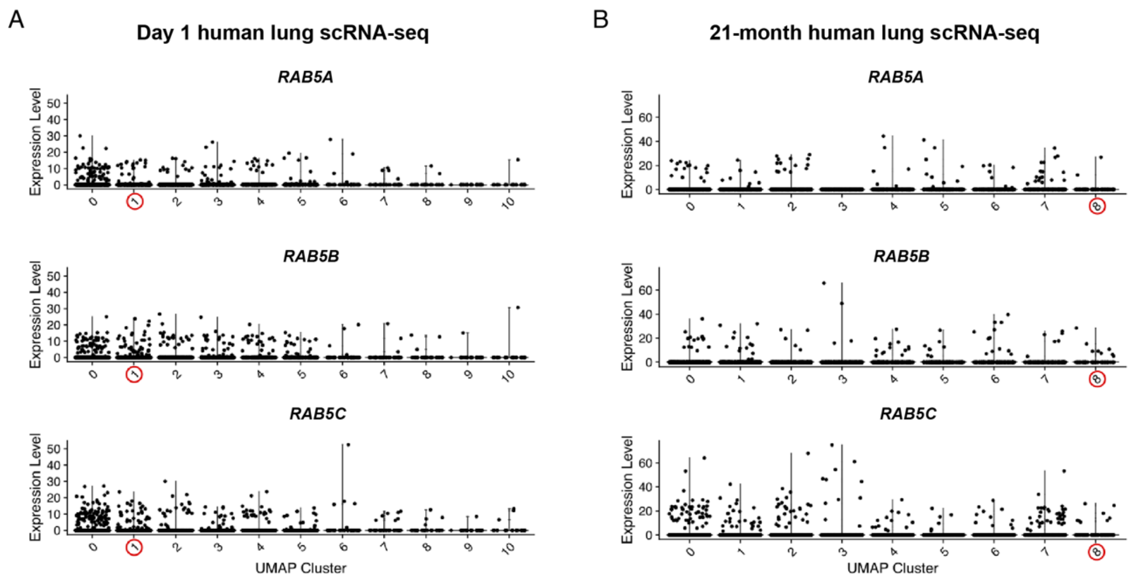

Fig S7. *RAB5A*, *RAB5B,* and *RAB5C* RNA expression levels across the lung single cell RNA-seq clusters. (A) *RAB5A*, *RAB5B,* and *RAB5C* RNA expression levels from day 1 human scRNA-seq clusters from Fig S5. Cluster 1 (red circles) marks putative AT2 cells. (B) *RAB5A*, *RAB5B,* and *RAB5C* RNA expression levels from 21-month scRNA-seq clusters from Fig S6. Cluster 8 (red circles) marks putative AT2 cells.

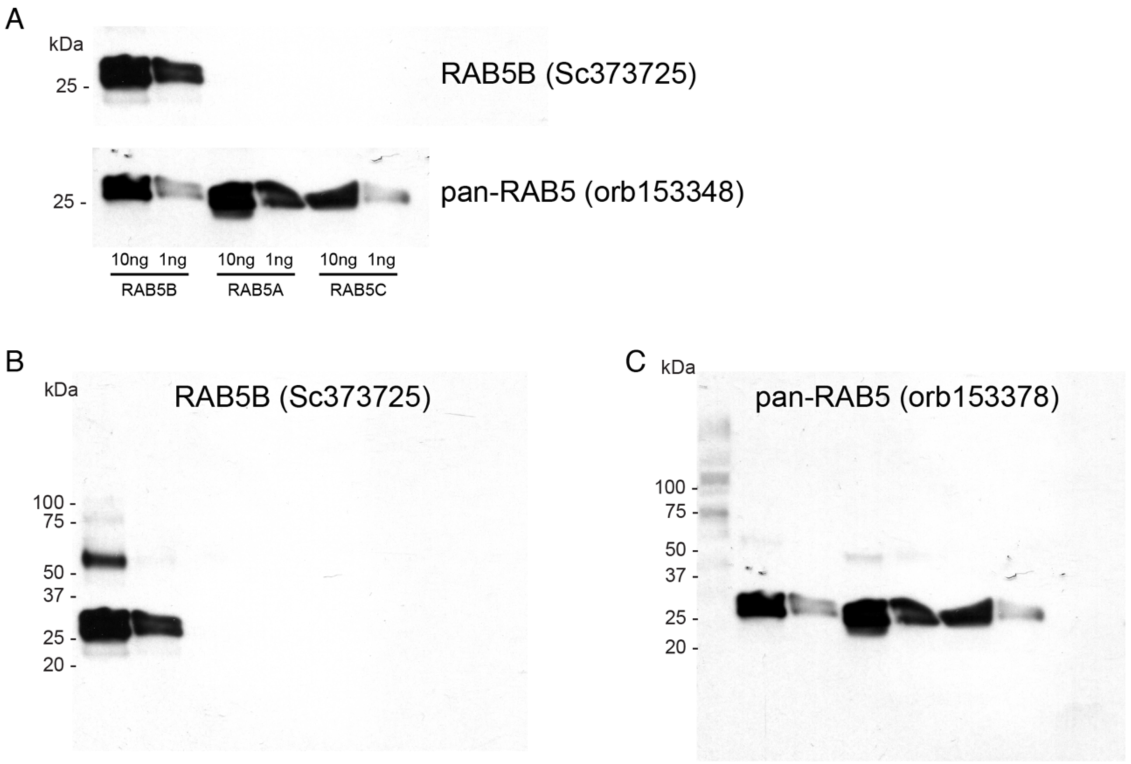

Fig S8. Specificity of the anti-RAB5B and anti-total (pan) RAB5 antibodies. (A–C) Western blots of 10-fold dilutions of pure human RAB5B (left), RAB5A (middle), and RAB5C (right) proteins that were blotted with the anti-RAB5B antibody SC373725 (A, upper and B) and anti-total (pan) RAB5 antibody orb153348 (A, lower and C) (See **Table S4**). (A, upper and lower) shows only the RAB5 region of the gel around 25kD. (B) and (C) show the full gels with bands included in panel A. The anti-RAB5B antibody does not cross react with excess RAB5A and RAB5C. The anti-total (pan) RAB5 antibody reacts with all the RAB5 paralog proteins. Proteins were separated on a 4-12% Bis-Tris gel.

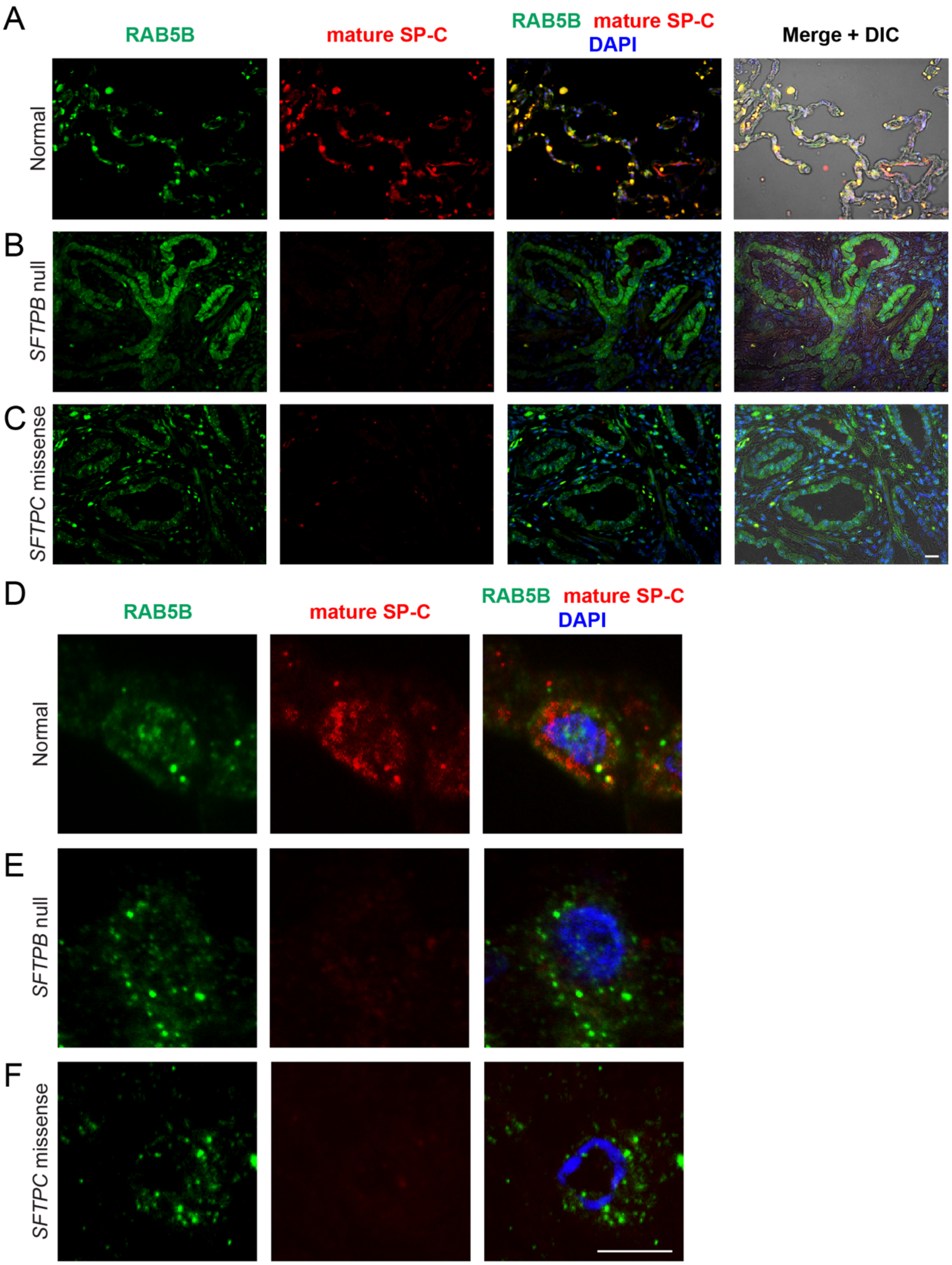

Fig S9. RAB5B and mature SP-C staining in lung sections from normal donor or individuals with disorders of surfactant dysfunction. Lung sections from normal donor or individuals with *SFTPB* homozygous null, or *SFTPC* heterozygous missense variants were stained for RAB5B, mature SP-C, and DNA, with DAPI. (A–C) Low magnification (scale bar, 20 μm) view of lung tissue,

including differential interference contrast microscopy (DIC) overlay. (D–F) High magnification (scale bar, 5 μm) view of single cells. Mature SP-C staining was not observed in *SFTPB* null and *SFTPC* missense cells, demonstrating specificity of the antibody for mature SP-C. At high magnification, RAB5B staining was similar between the normal and the *SFTPB* null and the *SFTPC* missense variant cells (D–F). However, due to AT2 cell hyperplasia, RAB5B staining appears more extensive in the low magnification images of the *SFTPB* null and the *SFTPC* missense variant lung tissues (B, C).

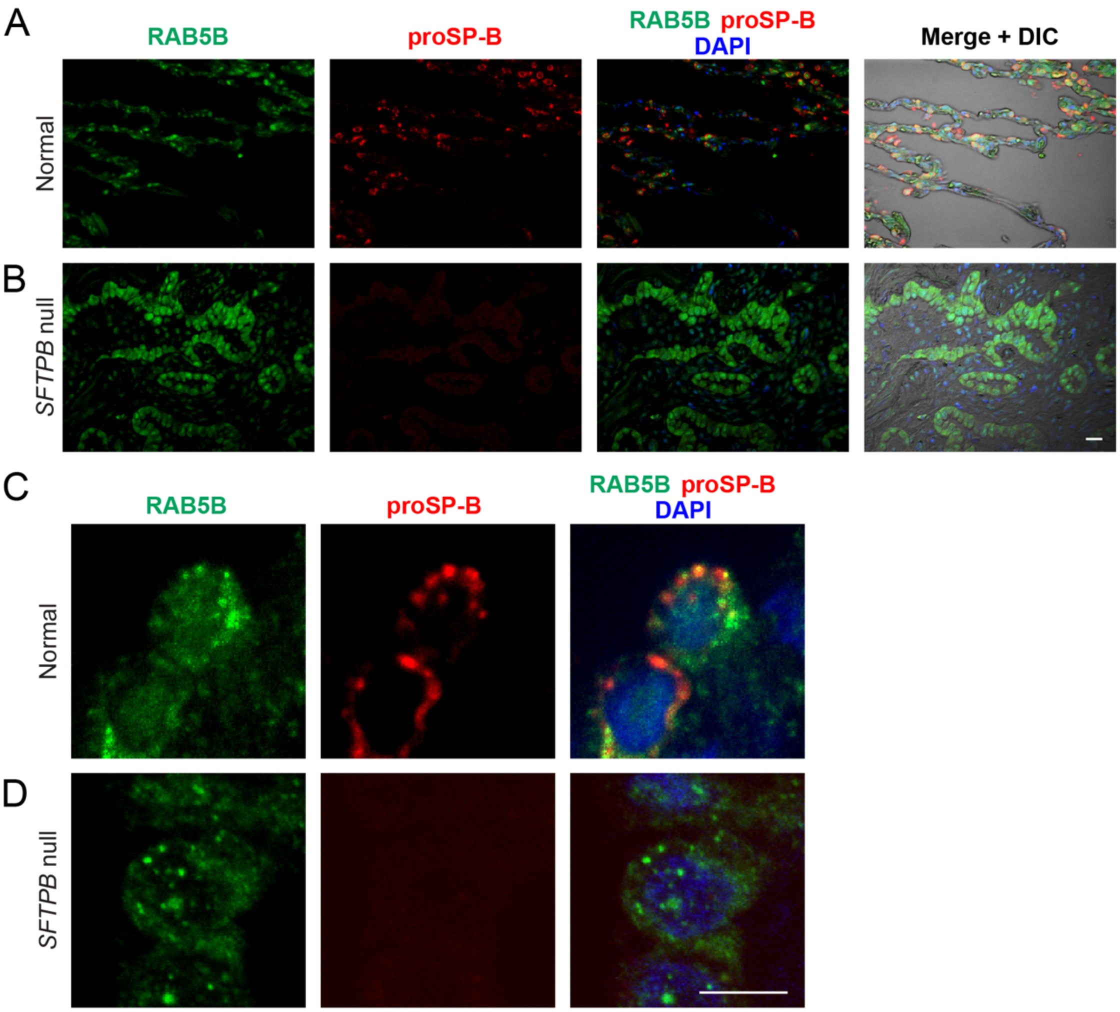

Fig S10. RAB5B and proSP-B staining in lung sections from normal donor and an infant homozygous for *SFTPB* null variant. Lung sections from normal donor and infant homozygous for *SFTPB* null variant were stained for RAB5B, proSP-B, and DNA, with DAPI. (A and B) Low magnification (scale bar, 20 μm) view of lung tissue, including differential interference contrast microscopy overlay (right). (C and D) High magnification (scale bar, 5 μm) view of single cells. ProSP-B staining was not observed in *SFTPB* null cells, demonstrating specificity of the antibody for proSP-B. At the cellular level, RAB5B staining was similar between the normal and the *SFTPB* null (C, D). However, due to AT2 cell hyperplasia, RAB5B staining appears more extensive in the low magnification images of the *SFTPB* null (B).

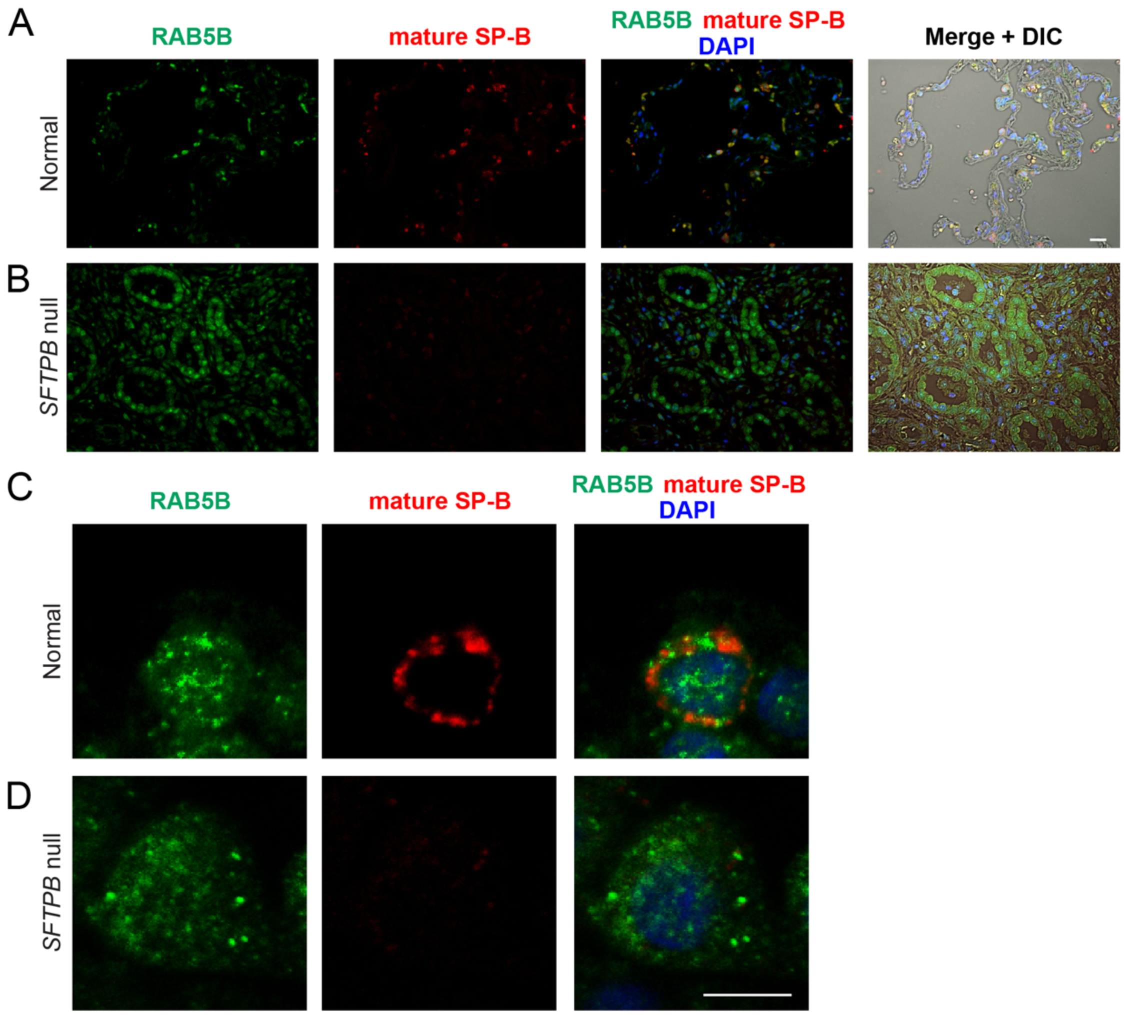

Fig S11. RAB5B and mature SP-B staining in lung sections from a normal donor and an infant homozygous for *SFTPB* null variant. Lung sections from normal donor and an infant homozygous for *SFTPB* null variant were stained for RAB5B, mature SP-B, and DNA, with DAPI. (A and B) Low magnification (scale bar, 20 μm) view of lung tissue, including differential interference contrast microscopy overlay (right). (C and D) High magnification (scale bar, 5 μm) view of single cells. Mature SP-B staining was not observed in the *SFTPB* null cells, demonstrating specificity of the antibody for mature SP-B. At the cellular level, RAB5B staining was similar between the normal and the *SFTPB* null. However, due to AT2 cell hyperplasia, RAB5B staining appears more extensive in the low magnification images of the *SFTPB* null (B).

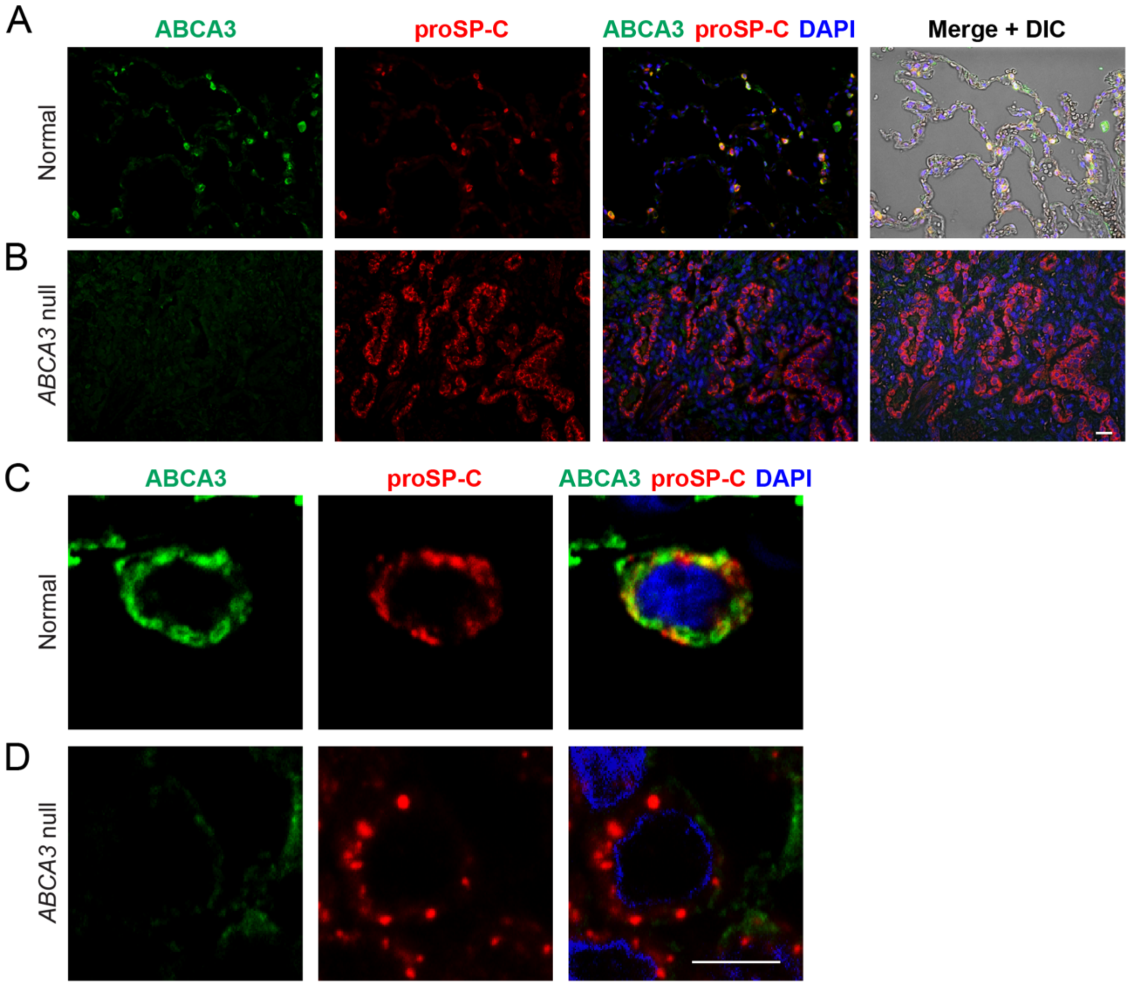

Fig S12. ABCA3 and proSP-C staining in lung sections from normal donor and an infant compound heterozygous for *ABCA3* null variants. Lung sections from normal donor and an infant compound heterozygous for *ABCA3* null variant were stained for RAB5B, ABCA3, and DNA, with DAPI. (A and B) Low magnification (scale bar, 20 μm) view of lung tissue, including differential interference contrast microscopy overlay (right). (C and D) High magnification (scale bar, 5 μm) view of single cells. ABCA3 staining was not observed in *ABCA3* null cells, demonstrating specificity of the antibody for ABCA3.

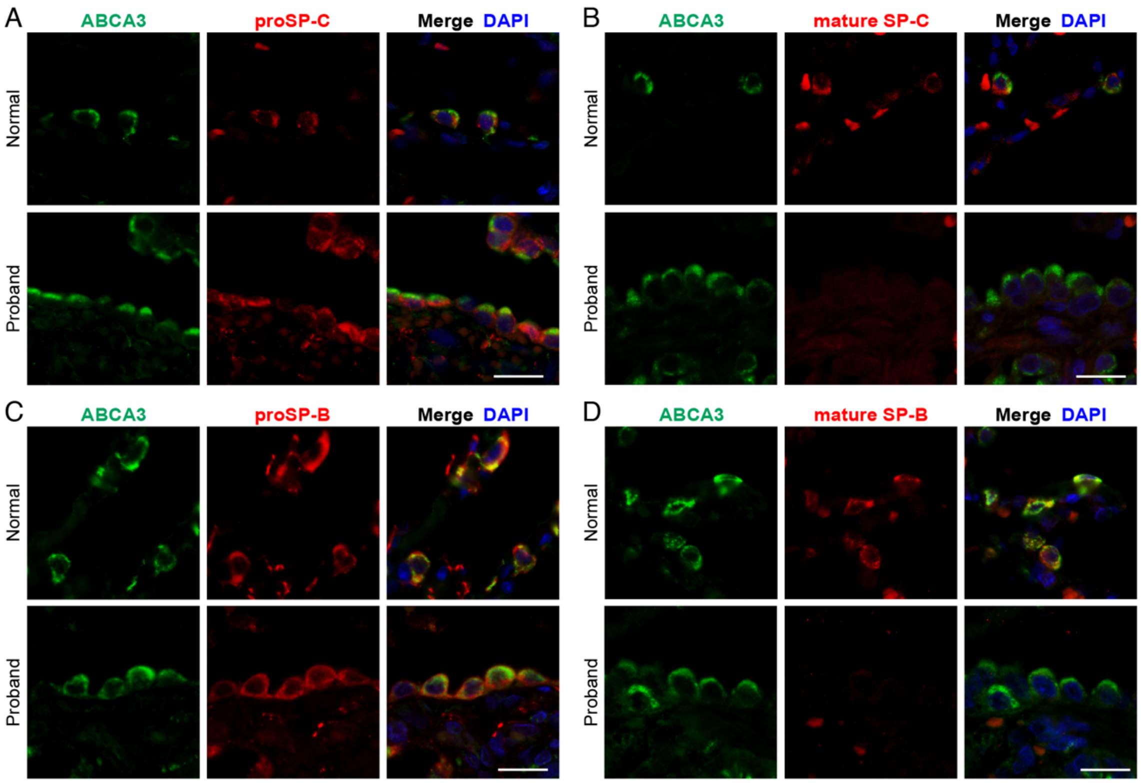

Fig S13. ABCA3, proSP-C, proSP-B and mature SP-C and SP-B staining in lung sections from normal donor and the proband. Lung sections from normal donor and the proband were stained for ABCA3 and proSP-C (A), ABCA3 and mature SP-C (B), ABCA3 and proSP-B (C), and ABCA3 and mature SP-B, and DNA, with DAPI. Intermediate magnification view (scale bar, 20 μm). ABCA3 staining is essentially identical between the normal and the proband, with the proband having increased number of AT2 cells and staining more restricted to the external surface. (B) and (D) show that proband AT2 cells, as define by expression of ABCA3, have a significant reduction in mature SP-C and SP-B staining.

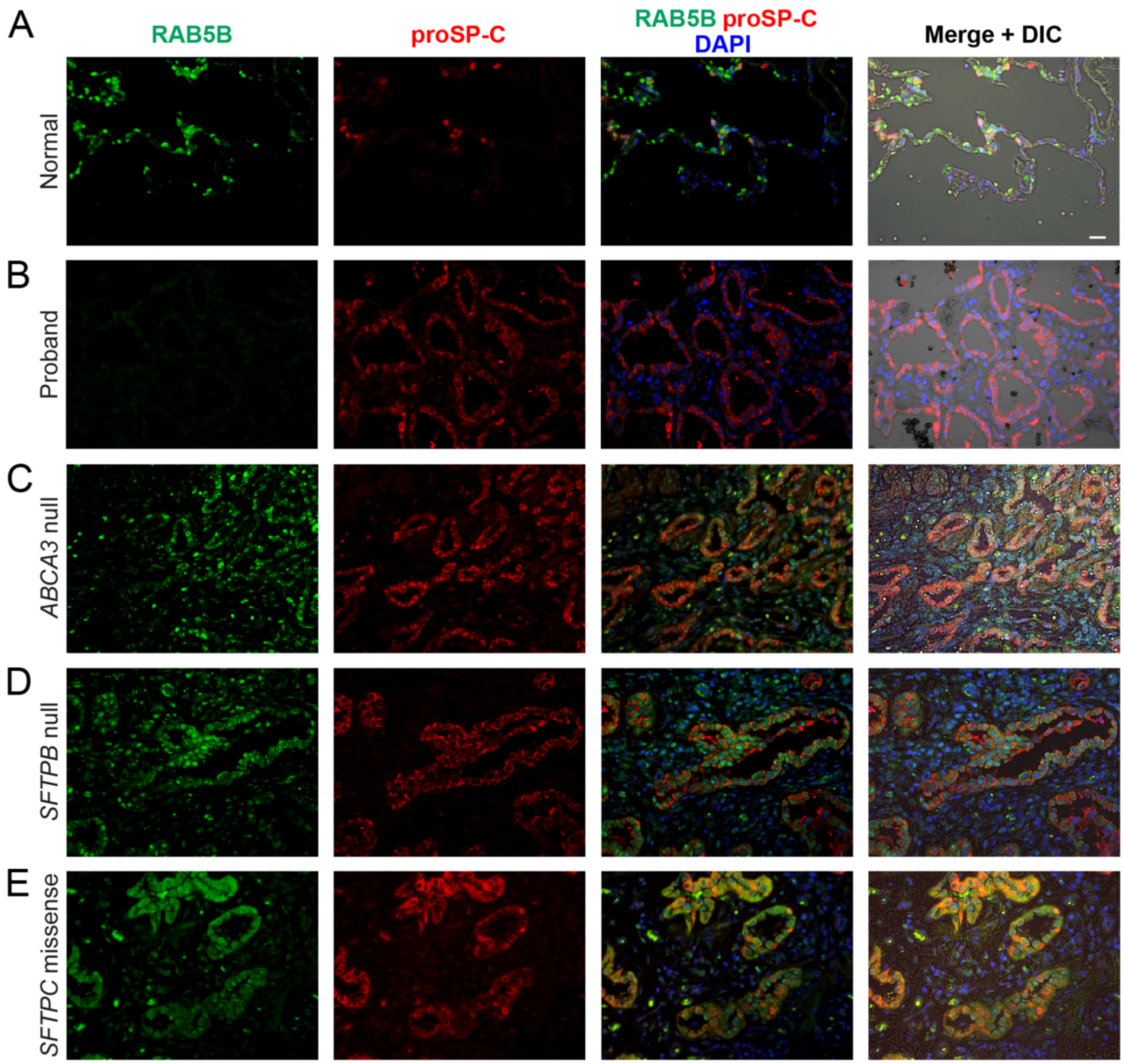

Fig S14. RAB5B and proSP-C staining in lung sections from normal donor and individuals with disorders of surfactant dysfunction. Lung sections from normal donor (A), the proband (B), *ABCA3* null homozygous (C), *SFTPB* null homozygous (D), and *SFTPC* missense heterozygous (E) individuals stained for RAB5B, proSP-C, and DNA. Low magnification (scale bar, 20 μm) view of lung tissue, including differential interference contrast microscopy overlay (right). The RAB5B staining appears more extensive among the 3 individuals with disorders of surfactant dysfunction (C–E) as compared to the normal due to AT2 cell hyperplasia.

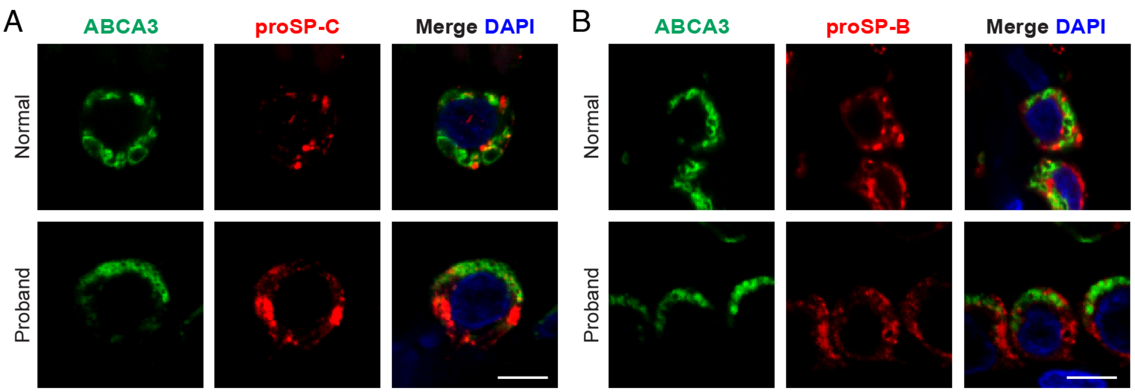

Fig S15. ABCA3 and proSP-C or proSP-B staining in lung sections from normal donor and the proband.
Lung sections from normal donor (upper panels) and the proband (lower panels) were stained for ABCA3 and proSP-C (A) or ABCA3 and proSP-B (B), and DAPI. High magnification (scale bar, 5 μm) view of single AT2 cell. The proSP-C and proSP-B staining appear similar between the normal and the proband.

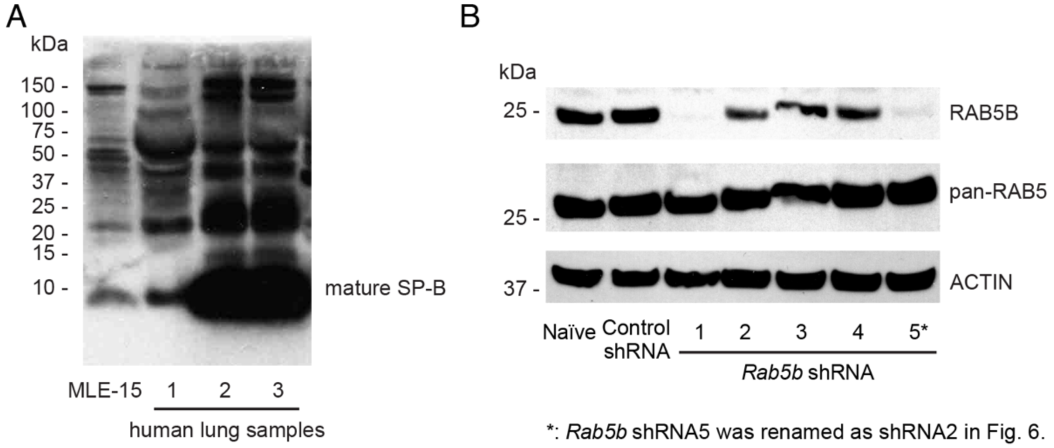

Fig S16. Western blot of MLE-15 cells and normal donor lung samples showing mature SP-B and the effect of different *Rab5b* shRNAs on RAB5B and total RAB5 protein levels. (A) Western blot of MLE-15 (left) and lysates from three normal donor lung samples 1, 2, and 3 (right) reacted with anti-mature SP-B antibody (full gel). Position of mature SP-B, ~9kD, is indicated. (B) Western blots showing the effect of shRNA treatment on RAB5B (top), total (Pan) RAB5 (middle), and ACTIN (bottom) proteins from MLE-15 cell lysates. Lanes, from left to right, Naïve MLE-15 cells, control shRNA, mouse *Rab5b* shRNAs 1 through 5. RAB5B protein is significantly knocked down in shRNAs 1 and 5 (renamed as shRNA2 in Figure 6), while total RAB5 protein, including RAB5A and RAB5C, is largely unaffected.

SI References

1. Lupski JR*, et al.* (2013) Exome sequencing resolves apparent incidental findings and reveals further complexity of *SH3TC2* variant alleles causing Charcot-Marie-Tooth neuropathy. *Genome Med* 5(6):57.
2. Karczewski KJ*, et al.* (2020) The mutational constraint spectrum quantified from variation in 141,456 humans. *Nature* 581(7809):434-443.
3. Fromer M & Purcell SM (2014) Using XHMM Software to Detect Copy Number Variation in Whole-Exome Sequencing Data. *Curr Protoc Hum Genet* 81:7 23 21-21.
4. Geoffroy V*, et al.* (2018) AnnotSV: an integrated tool for structural variations annotation. *Bioinformatics* 34(20):3572-3574.
5. Paix A, Folkmann A, Rasoloson D, & Seydoux G (2015) High Efficiency, Homology-Directed Genome Editing in *Caenorhabditis elegans* Using CRISPR-Cas9 Ribonucleoprotein Complexes. *Genetics* 201(1):47-54.
6. Dokshin GA, Ghanta KS, Piscopo KM, & Mello CC (2018) Robust Genome Editing with Short Single-Stranded and Long, Partially Single-Stranded DNA Donors in *Caenorhabditis elegans*. *Genetics* 210(3):781-787.
7. Arribere JA*, et al.* (2014) Efficient marker-free recovery of custom genetic modifications with CRISPR/Cas9 in *Caenorhabditis elegans*. *Genetics* 198(3):837-846.
8. Frokjaer-Jensen C*, et al.* (2008) Single-copy insertion of transgenes in *Caenorhabditis elegans*. *Nat Genet* 40(11):1375-1383.
9. Dickinson DJ, Pani AM, Heppert JK, Higgins CD, & Goldstein B (2015) Streamlined Genome Engineering with a Self-Excising Drug Selection Cassette. *Genetics* 200(4):1035-1049.
10. Roussel N, Sprenger J, Tappan SJ, & Glaser JR (2014) Robust tracking and quantification of *C. elegans* body shape and locomotion through coiling, entanglement, and omega bends. *Worm* 3(4):e982437.
11. Patro R, Duggal G, Love MI, Irizarry RA, & Kingsford C (2017) Salmon provides fast and bias-aware quantification of transcript expression. *Nat Methods* 14(4):417-419.
12. Poteryaev D, Fares H, Bowerman B, & Spang A (2007) *Caenorhabditis elegans* SAND-1 is essential for RAB-7 function in endosomal traffic. *EMBO J* 26(2):301-312.
13. Kamath RS & Ahringer J (2003) Genome-wide RNAi screening in *Caenorhabditis elegans*. *Methods* 30(4):313-321.
14. Mohammad A*, et al.* (2018) Initiation of Meiotic Development Is Controlled by Three Post-transcriptional Pathways in *Caenorhabditis elegans*. *Genetics* 209(4):1197-1224.
15. Ardini-Poleske ME*, et al.* (2017) LungMAP: The Molecular Atlas of Lung Development Program. *Am J Physiol Lung Cell Mol Physiol* 313(5):L733-L740.
16. Stuart T & Satija R (2019) Integrative single-cell analysis. *Nat Rev Genet* 20(5):257-272.
17. Whitsett JA, Wert SE, & Weaver TE (2015) Diseases of pulmonary surfactant homeostasis. *Annu Rev Pathol* 10:371-393.

18. Nogee LM, de Mello DE, Dehner LP, & Colten HR (1993) Brief report: deficiency of pulmonary surfactant protein B in congenital alveolar proteinosis. *N Engl J Med* 328(6):406-410.
19. Alysandratos KD*, et al.* (2021) Patient-specific iPSCs carrying an *SFTPC* mutation reveal the intrinsic alveolar epithelial dysfunction at the inception of interstitial lung disease. *Cell Rep* 36(9):109636.
20. Horani A, Nath A, Wasserman MG, Huang T, & Brody SL (2013) Rho-associated protein kinase inhibition enhances airway epithelial Basal-cell proliferation and lentivirus transduction. *Am J Respir Cell Mol Biol* 49(3):341-347.
21. Schindelin J*, et al.* (2012) Fiji: an open-source platform for biological-image analysis. *Nat Methods* 9(7):676-682.
22. Yoshimura J*, et al.* (2019) Recompleting the *Caenorhabditis elegans* genome. *Genome Res* 29(6):1009-1022.
23. Smurova K & Podbilewicz B (2016) RAB-5- and DYNAMIN-1-Mediated Endocytosis of EFF-1 Fusogen Controls Cell-Cell Fusion. *Cell Rep* 14(6):1517-1527.
24. Dejima K*, et al.* (2018) An Aneuploidy-Free and Structurally Defined Balancer Chromosome Toolkit for *Caenorhabditis elegans*. *Cell Rep* 22(1):232-241.
25. Fares H & Greenwald I (2001) Genetic analysis of endocytosis in *Caenorhabditis elegans*: coelomocyte uptake defective mutants. *Genetics* 159(1):133-145.
26. Balklava Z, Pant S, Fares H, & Grant BD (2007) Genome-wide analysis identifies a general requirement for polarity proteins in endocytic traffic. *Nat Cell Biol* 9(9):1066-1073.
